# Utility of entomological indices for predicting transmission of dengue virus: secondary analysis of data from the Camino Verde trial in Mexico and Nicaragua

**DOI:** 10.1371/journal.pntd.0008768

**Published:** 2020-10-26

**Authors:** Arcadio Morales-Pérez, Elizabeth Nava-Aguilera, Carlos Hernández-Alvarez, Víctor Manuel Alvarado-Castro, Jorge Arosteguí, José Legorreta-Soberanis, Miguel Flores-Moreno, Liliana Morales-Nava, Eva Harris, Robert J. Ledogar, Neil Andersson, Anne Cockcroft

**Affiliations:** 1 Centro de Investigación de Enfermedades Tropicales (CIET), Universidad Autónoma de Guerrero, Acapulco, Guerrero, México; 2 CIETinternational in Nicaragua, Managua, Nicaragua; 3 Division of Infectious Diseases and Vaccinology, School of Public Health, University of California, Berkeley, United States of America; 4 CIETinternational, New York, United States of America; 5 Department of Family Medicine, McGill University, Montreal, Canada; University of California San Francisco, UNITED STATES

## Abstract

Dengue vector entomological indices are widely used to monitor vector density and disease control activities. But the value of these indices as predictors of dengue infection is not established. We used data from the impact assessment of a trial of community mobilization for dengue prevention (*Camino Verde*) to examine the associations between vector indices and evidence of dengue infection and their value for predicting dengue infection levels. In 150 clusters in Mexico and Nicaragua, two entomological surveys, three months apart, allowed calculation of the mean Container Index, Breteau index, Pupae per Household Index, and Pupae per Container Index across the two surveys. We measured recent dengue virus infection in children, indicated by a doubling of dengue antibodies in paired saliva samples over the three-month period. We examined the associations between each of the vector indices and evidence of dengue infection at household level and at cluster level, accounting for trial intervention status. To examine the predictive value for dengue infection, we constructed receiver operating characteristic (ROC) curves at household and cluster level, considering the four vector indices as continuous variables, and calculated the positive and negative likelihood ratios for different levels of the indices. None of the vector indices was associated with recent dengue infection at household level. The Breteau Index was associated with recent infection at cluster level (Odds ratio 1.36, 95% confidence interval 1.14–1.61). The ROC curve confirmed the weak predictive value for dengue infection of the Breteau Index at cluster level. Other indices showed no predictive value. Conventional vector indices were not useful in predicting dengue infection in Mexico and Nicaragua. The findings are compatible with the idea of sources of infection outside the household which were tackled by community action in the *Camino Verde* trial.

## Author summary

Vector control is the basis of preventing dengue virus infection. Measurement of dengue vector indices is widely used to monitor dengue control activities, but their utility as predictors of dengue infection is not clear. We used data about vector indices and evidence of recent dengue virus infection from a trial of community mobilization for dengue prevention in Mexico and Nicaragua (*Camino Verde*), to examine how four standard vector indices could predict dengue infection. Only the Breteau index was associated with evidence of dengue infection, and that only at cluster level. Receiver operating characteristic (ROC) curves confirmed the Breteau Index was only a weak predictor of infection at cluster level; none of the other indices had predictive value. These findings indicate that the four entomological indices studied were not useful predictors of dengue infection. The results are compatible with the idea of sources of infection outside the household which were tackled by community action in the *Camino Verde* trial.

## Introduction

The incidence of dengue fever has increased 30-fold in the last five decades [1]. It is estimated that there are 390 million dengue infections in the world each year, of which 96 million have clinical manifestations [2]. Mass vaccination for dengue with currently available vaccines is problematic [3], and vector control remains the mainstay of dengue prevention [1].

The main vector of dengue, *Aedes aegypti*, inhabits urban and suburban areas [4]. It also transmits yellow fever, chikungunya and zika [5,6,7]. The main *Aedes aegypti* breeding sites are containers for storing water for domestic use and receptacles that accumulate water incidentally [8,9].

The World Health Organization recommends the use of entomological indices to monitor vector density and evaluate control actions [10]. Most trials of interventions for dengue control have reported impact on entomological indices, in particular the classical S*tegomyia* indices combining larval and pupal data, with relatively few reporting impact on clinical dengue cases or serological evidence of dengue infection [11]. Pupal indices [12,13,14] have been proposed as a better indicator of adult vector density [15,16,17,18].

Although vector indices, most commonly those measuring combined larvae and pupae density, remain the most frequent method of surveillance for assessing the risk of dengue outbreaks [19], the association between entomological indices and risk of dengue transmission remains unclear. A 2014 systematic review included 18 studies of the association between vector indices and clinical dengue cases [20]. Eleven of the studies used combined larval and pupal indices, four sampled only larvae, two reported pupal indices, and three sampled adult mosquitoes. Study methodology was generally weak. Four of 13 studies reported a significant relationship between vector indices and clinical cases of dengue, in five the evidence was ambiguous, and four studies reported no evidence of association [20]. In a recent analysis of data from Peru, Cromwell and colleagues examined the associations between a wide range of vector indices (including measures of adult mosquitoes) and serological evidence of dengue infection; they reported significant associations between longitudinal vector density measurements, but not cross-sectional measurements, and dengue infection [21].

Even when there is association between vector indices and dengue infection, this does not necessarily mean that higher indices are useful predictors of the risk of dengue infection. We used data from the impact survey of the *Camino Verde* trial of evidence-based community mobilization for dengue prevention conducted in Mexico and Nicaragua [22] to examine the association between several vector indices and serological evidence of recent dengue infection in children. The objectives of our study are to: examine the association between vector indices and serological dengue infection; assess the utility of the indices for predicting cases of recent serological infection; and examine whether associations or utility for prediction differed between intervention and control clusters in the trial.

## Methods

### Ethics statement

The Nicaraguan arm of the Camino Verde trial was approved by institutional review boards at the University of California, Berkeley (22 July 2010), the Nicaraguan Ministry of Health (25 August 2010), and CIETinternational (1 August 2010). The Mexican arm was approved by the CIETcanada research ethics board (16 November 2009) and the ethics committee of the Centro de Investigación de Enfermedades Tropicales at the Universidad Autónoma de Guerrero (27 November 2009). All boards performed annual review and approval throughout the study. Oral consent was obtained from an adult head of household for entomological surveys as approved by the institutional review boards. In all cases before obtaining a saliva sample, the parents or the guardian of the child gave their consent. All the committees approved the procedures for the collection of samples of saliva and data. Oral consent was obtained because many people do not know how to write and they distrust signing documents but they trust the word of mouth. Oral consent was recorded in the survey notebooks at the time households gave access to their homes.

### Context of this study

The *Camino Verde* trial methods and findings are described in detail elsewhere [22,23,24]. The trial took place in 90 clusters (of about 140 households each) in coastal regions of Guerrero state, Mexico, and in 60 clusters in Managua, Nicaragua, randomly allocated to intervention or control groups. The intervention comprised sharing of evidence from a baseline survey about mosquito breeding sites, household visits by community volunteers, and community and household actions to reduce mosquito breeding locations. Intervention clusters had significantly lower vector indices at follow-up (household index, container index, Breteau index, and pupae per person). They also had significantly fewer cases of self-reported dengue, and significantly fewer cases of recent dengue infection, measured by paired serology in children. Field teams collected entomological data twice in the impact measurement, three months apart, straddling the seroconversion interval. We compared entomological data (averaged across both measurements) as the exposure with serological evidence of dengue transmission as the outcome.

### Estimation of dengue infection

Fieldworkers collected paired saliva samples from children aged three to nine years for the detection of dengue-specific IgG antibodies. The first samples were at the peak of the dengue season (between August and September 2012) and the second samples, about three months later, were at the end of the dengue season (between November 2012 and January 2013). Some households did not have children in the relevant age group and in others the parents did not agree to a saliva sample being taken from their child(ren). In our analysis at household level, we included only those households which provided paired saliva samples from at least one child, counting a household as “positive” for recent dengue infection if at least one child had serological evidence of recent infection. In our analysis at cluster level, our outcome measure was the proportion of households in the cluster with at least one child with serological evidence of dengue infection, out of the households with children who had serological testing.

We used an ELISA procedure to detect dengue-specific IgG antibodies in the paired saliva samples [25,26]. Specialized software automatically transferred optical densities from the ELISA reader to the computer, calculated IgG units, and verified the data [27]. We considered a doubling of dengue-specific IgG between the first and second saliva sample to be evidence of recent dengue infection.

We considered that a child had infection in the first or second saliva sample when the ELISA result showed there were 15 units of IgG or more.

### Entomological indices

Fieldworkers inspected all water containers in and around all houses in each cluster, and interviewed the householders. They recorded the numbers and types of containers and collected all larvae and pupae present into labelled bags, which were transported to the laboratory and stored at -20°C until examination by expert entomologists. Entomologists identified and quantified larvae and pupae, using an Olympus CS41 stereoscopic microscope and taxonomic keys [28,29]. They recorded any adult exuvia (exoskeleton remains) or mosquitoes found in the samples as pupae.

We calculated the following indices: container index (CI) (number of containers with at least one larva or pupa divided by the total number of containers inspected); Breteau index (BI) (number of containers with at least one larva or pupa divided by the total number of households inspected); pupae per household index (PHI) (total number of pupae found divided by the total number of households inspected); pupae per container index (PCI) (total number of pupae found divided by the total number of containers inspected).

To examine associations with and predictive utility of entomological data, we averaged the indices measured at the beginning and end of the three-month period in each household. Some households did not participate in the entomological survey at either the first or the second time-point; in our analysis we included only those households that had entomological data from both time points.

### Statistical analysis

Analysis relied mainly on CIETmap open-source software [30], which provides an interface with the R programming language [31].

### Estimating associations between entomological indices and dengue infection

We examined associations between the indices and evidence of infection, at household level and at cluster level. We undertook an analysis to see how stable our entomological data were in time and space. We also conducted a subgroup analysis in which we excluded households that had at least one infected child at the first measurement point, and a further analysis in which we excluded households with any self-reported case of dengue.

*At household level*: Treating the entomological indices as categorical exposure variables, with categories of 0 and >0, we used the Mantel-Haenszel procedure [32], to estimate the odds ratio (OR) and 95% confidence interval, adjusted for clustering (95% CIca) by the Lamothe method [33], with stratification by intervention status of each cluster. We tested the significance of any differences in the associations between intervention and control clusters [34]. We also did a further household level analysis considering the indices as continuous variables, using Kendall’s range correlation test [35].

*At cluster level*: We categorized the infection status of each cluster as being above or below the mean proportion of households with positive dengue serology across all clusters. For each vector index, we undertook a gamma regression of the mean index for each cluster (exposure variable) against the infection status of the cluster (outcome variable). We included the intervention status of the clusters as another variable in each model.

### Estimating utility of entomological variables for predicting dengue infection

Treating the vector indices as diagnostic tests and evidence of recent dengue infection as presence of a condition, we estimated the utility of the vector indices for predicting dengue infection in two ways. We created Receiver Operating Characteristic (ROC) curves [36,37] using SPSS, treating the vector indices as continuous variables [38]. We calculated the area under the curve (AUC) as an overall indicator of diagnostic accuracy. The AUC can have any value between 0 and 1. A perfect diagnostic test has an AUC of 1. An excellent test has AUC values between 0.9–1.0, a very good test values of 0.8–0.9, good 0.7–0.8, adequate 0.6–0.7, bad 0.5–0.6. A test is not useful when the AUC value is ≤0.5.

We also estimated the positive likelihood ratio (LR+ = sensitivity/(1-specificity)) and negative likelihood ratio (LR- = (1-sensitivity)/specificity) [39] and their confidence intervals [40] at certain cut-off points for each vector index. An LR+ of more than 10 is strong evidence of presence of the condition, and a LR- of less than 0.1 is strong evidence of the absence of the condition. At household level, we used the cut-off of 0 vs >0 for the value of each index. At cluster level, from the ROC curve for each vector index, we identified three cut-off points: an “optimum” point, with the highest sensitivity and a specificity of at least 50%; a high sensitivity point, with 80% sensitivity regardless of the specificity; and a high specificity point, with 80% specificity, regardless of the sensitivity.

## Results

A total of 6607 households across the 150 clusters provided paired saliva samples from at least one child in the impact survey. Very few households (58) refused to allow saliva samples to be taken from their children. Among the households providing paired saliva samples, 6440 participated in the entomological survey at the two time-points (coinciding with the first and second saliva samples). Again, very few households (109) refused to participate in the entomological survey.

The seroconversion proportion at cluster level was 11.3%: 10.4% in intervention clusters and 12.3% in control clusters. At household level the seroconversion proportion was 13%; 11.3% in intervention sites and 14.8% in control sites. S1, S2, S3 and S4 Figs show the frequency distributions of the measured ranges of the four indices used in this analysis. The levels of the indicators measured at the two times points were strongly correlated (S1 Table).

Some 4.5% (288/6415) of households had at least one seropositive child (with 15 IgG or more units) in the initial measurement: 4.7% (155/3306) of households in intervention communities and 4.3% (133/3109) of households in control communities. We found no association between households with at least one child infected with dengue virus in the first measurement and initial entomological indices (S2 Table).

### Association between vector indices and dengue infection

#### At household level

None of the vector indices was associated with the presence of recent dengue infection. As shown in Table 1, the adjusted odds ratios for all the vector indices were close to 1.00. These odds ratios took into account the effect of the trial intervention, by stratification, and the test for heterogeneity between strata indicated that the lack of association was consistent between intervention and control clusters.

**Table 1:** Associations between vector indices and serological evidence of recent dengue infection in children at household level.

Index	Value	Fraction (%) households with dengue infection serology:		ORa (95% CIca)
		Negative	Positive^1^	
BI	0	4303/4927(87.3)	624/4927(12.7)	1.08 (0.92–1.27)
	>0	1301/1513(86)	212/1513(14)	
CI	0	4303/4927(87.3)	624/4927(12.7)	1.08 (0.92–1.27)
	>0	1301/1513(86)	212/1513(14)	
PCI	0	5065/5812(87.1)	747/582(12.9)	1.06 (0.84–1.34)
	>0	539/628(85.8)	89/628(14.2)	
PHI	0	5065/5812(87.1)	747/582(12.9)	1.06 (0.84–1.34)
	>0	539/628(85.8)	89/628(14.2)	

1 Positive dengue infection serology means at least one child aged 3–9 years old in the household had a doubling of dengue specific antibodies in paired saliva samples

ORa = odds ratio, adjusted for intervention status of the cluster

95%CIca = 95% confidence interval of OR, adjusted for clustering

The subgroup analysis excluding households with at least one infected child at the first measurement point yielded findings similar to those from the main analysis including all the households (S3 Table). Among households with at least one child with positive serology, 52.2% (435/833) had a single child with positive serology, 33.5% (279/833) two positive children, 12% (100/833) three positive children, 1.4% (12/833) four positive children and 0.8% (7/833) more than four.

Considering the entomological indices as continuous variables, there was no statistically significant association between the indices and serological evidence of recent dengue infection. (S4 Table). After excluding households that reported at least one case of dengue disease, the entomological indices did not change (S5 Table) and there was still no association between entomological indices and serological evidence of dengue infection (S6 Table).

#### At cluster level

There was a significant association between the Breteau Index in the cluster and the cluster having a higher proportion of households with positive dengue serology. There was no significant association between the mean value of any of the other vector indices in a cluster and the infection status of the cluster (Tables 2 and 3).

**Table 2:** Associations between vector indices and proportion of households with positive dengue serology at cluster level (150 clusters) from gamma regression.

Index	OR^1^ for association with infection status^2^ of cluster	95% CI^1^
Breteau Index	1.36	1.14–1.61
Mean container index	1.19	0.52–2.75
Mean pupae per container index	0.87	0.74–1.03
Mean pupae per household index	0.98	0.93–1.04

1 The OR and 95% CI take into account the effect of intervention status of each cluster

2 Clusters categorized as above or below mean proportion of households with positive dengue serology in children

**Table 3:** Associations between vector indices and proportion of households with positive dengue serology at cluster level (150 clusters) from Pearson’s linear regression.

Index	R	R^2^	P
Breteau Index	0.31	0.096	0.001
Mean container index	0.05	0.002	0.55
Mean pupae per container index	0.014	0.0001	0.87
Mean pupae per household index	0.118	0.014	0.15

**Fig 1:**
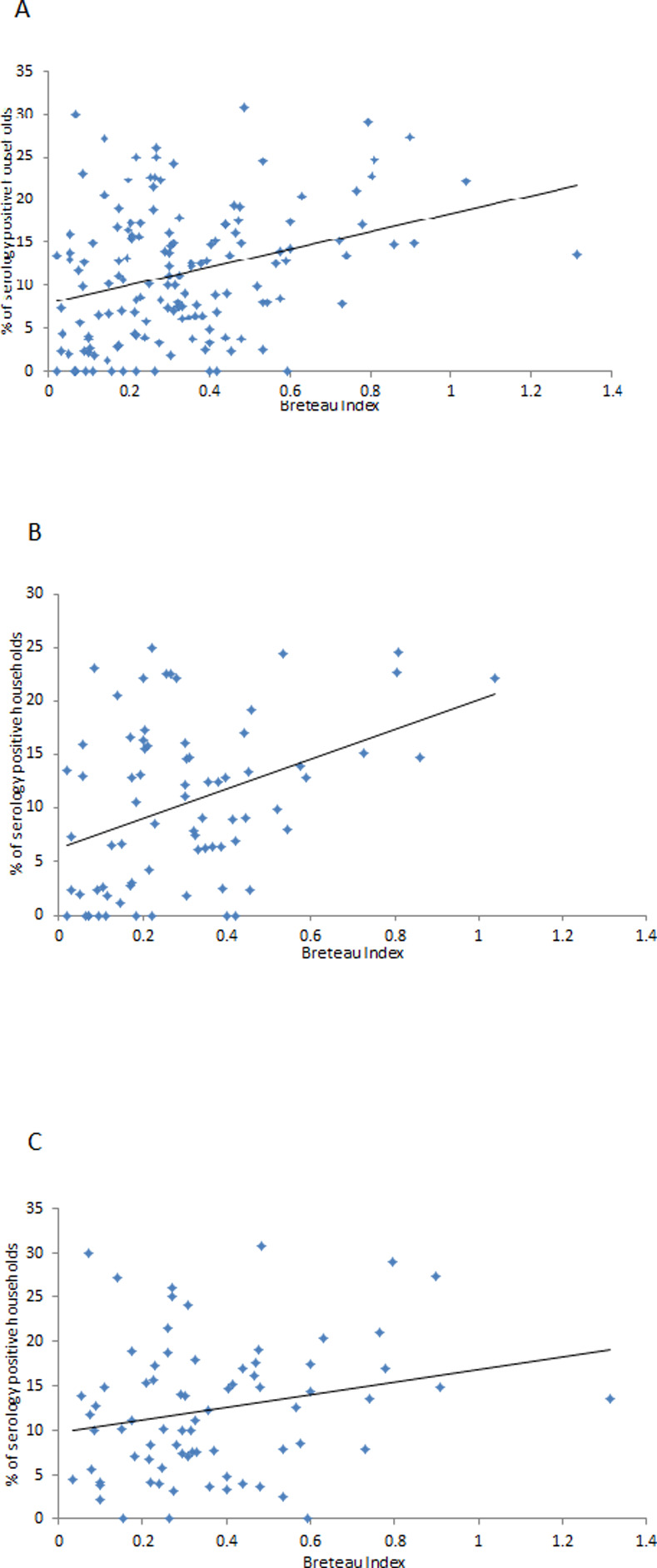
Breteau Index and proportion of serology positive households in clusters. In the scatter plots, each point represents a cluster. The vertical axis is the proportion of households in the cluster with at least one child with a doubling of dengue specific antibodies between paired saliva samples. The horizontal axis is the value of the Breteau Index in the cluster, averaged across two measurements three months apart. A. Scatter plot of Breteau Index and proportion of dengue serology positive households in all 150 clusters. B. Scatter plot of Breteau Index and proportion of serology positive households in 75 trial intervention clusters. C. Scatter plot of Breteau Index and proportion of serology positive households in 75 trial control clusters.

Fig 1 illustrates the association between the Breteau Index for the cluster and the level of dengue infection in the cluster, for all clusters, and for intervention and control clusters separately. The associations with the other vector indices are shown in s S5, S6 and S7 Figs.

### Utility of vector indices for predicting dengue infection

#### At household level

The AUC from the ROC curve for all the vector indices was close to 0.5, or even less than 0.5, indicating that the vector indices were not at all useful as diagnostic tests to predict dengue infection (Table 4). We calculated the LR+ and LR- for each index at the cut-off point of 0 vs >0. The LR+ was well below 10 and the LR- was well above 0.1. If anything, the pupal indices were even worse than the container and Breteau indices as tests for predicting dengue infection (Table 4).

**Table 4:** Predictive utility of vector indices at household level.

	BI	CI	PCI	PHI
Area under curve^1^ (95% CI)	0.515 (0.494–0.536)	0.512 (0.490–0.533)	0.505 (0.484–0.526)	0.506 (0.485–0.527)
LR+ (95% CI)	1.38 (1.18–1.61)	1.09 (0.96–1.24)	1.05 (0.81–1.35)	1.15 (0.48–2.72)
LR- (95% CI)	0.94 (0.91–0.97)	0.99 (0.98–1.01)	1.0 (0.98–1.02)	1.0 (0.99–1.01)

1 From ROC curve

LR+ Positive likelihood ratio with cut-off of 0 vs >0 for each index

LR- Negative likelihood ratio with cut-off of 0 vs >0 for each index

The results were very similar to those in Table 4 when we created ROC curves and estimated LR+ and LR- values for households in intervention and control sites separately (S7 Table).

**Fig 2:**
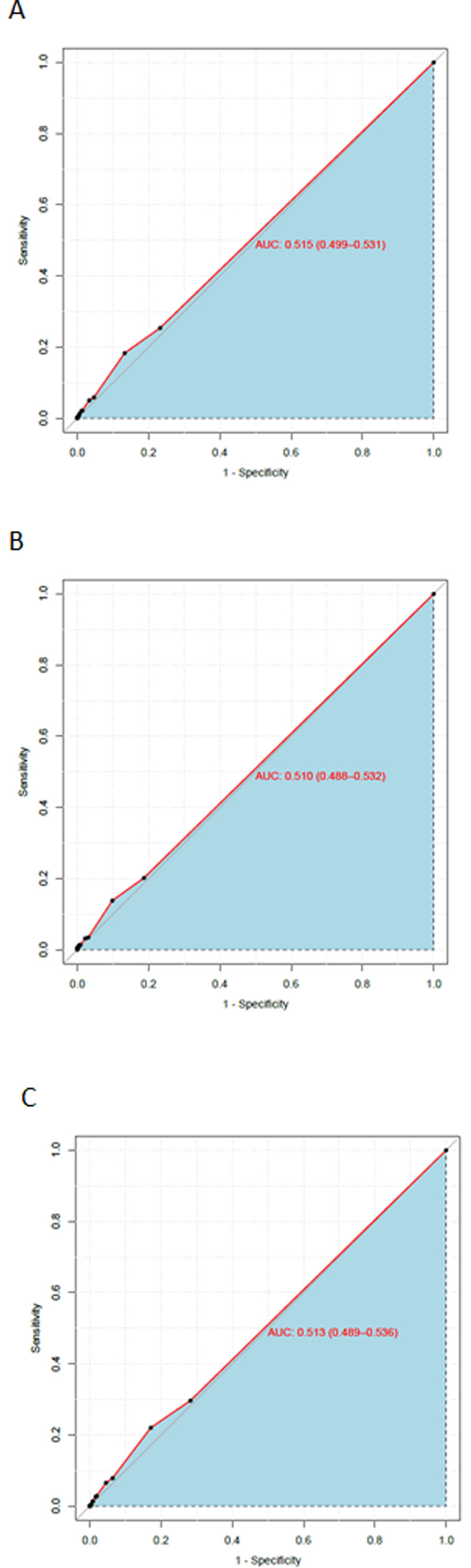
ROC curve for Breteau Index to predict dengue infection at household level. A. ROC curve for Breteau Index as a predictive test for dengue infection in households in all 150 clusters. B. ROC curve for Breteau Index as a predictive test for dengue infection in households in 75 trial intervention clusters. C. ROC curve for Breteau Index as a predictive test for dengue infection in households in 75 trial control clusters.

As an example, the ROC curve for the Breteau Index at household level is shown in Fig 2. The ROC curves for the other vector indices at household level, overall and in intervention and control sites separately, are shown in. S8, S9 and S10 Figs.

#### At cluster level

**Fig 3:**
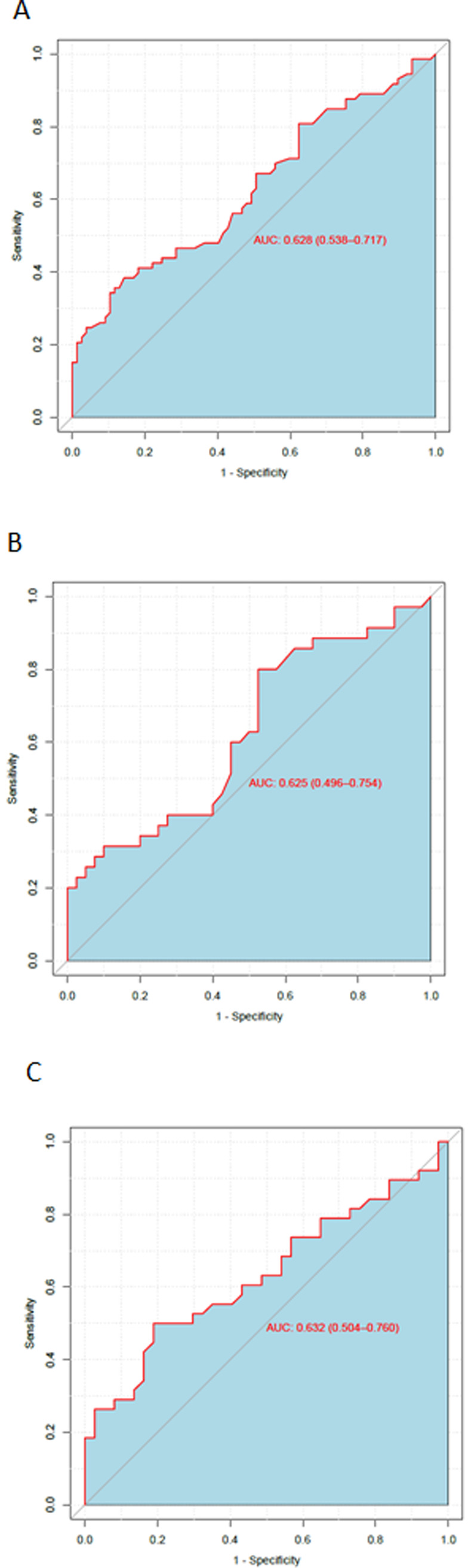
ROC curve for Breteau Index to predict dengue infection at cluster level. A. ROC curve for Breteau Index as a predictive test for dengue infection in all 150 clusters. B. ROC curve for Breteau Index as a predictive test for dengue infection in 75 trial intervention clusters. C. ROC curve for Breteau Index as a predictive test for dengue infection in 75 trial control clusters.

The AUC from the ROC curve for most of the indices was close to 0.5. For the Breteau index it was slightly better at 0.63, at the lower end of an adequate test. The ROC curve for the Breteau Index at cluster level is shown in Fig 3. The rest of the ROC curves at cluster level are shown in S11, S12 and S13 Figs.

Table 5 shows the AUC values for the four vector indices at cluster level, and the LR+ and LR- values calculated for the three cut-off points for each index. Even the Breteau index, with a higher AUC than the other vector indices, had LR+ and LR- values not close to the useful values of 10 (for LR+) or 0.1 (for LR-) at any cut-off point. The results were similar when we examined intervention and control clusters separately (S8 Table).

**Table 5:** Predictive utility of vector indices at cluster level.

	BI	CI	PCI	PHI
Area under curve^1^ (95% CI)	0.64 (0.54–0.72)	0.52 (0.43–0.62)	0.41 (0.32–0.50)	0.45 (0.36–0.54)
“Optimal” cut-off value^2^	0.33	0.072	0.0875	0.3575
LR+ (95% CI)	1.24 (0.94–1.63)	1.05 (0.76–1.46)	0.74 (0.53–1.03)	0.85 (0.60–1.18)
LR- (95% CI)	0.74 (0.50–1.09)	0.95 (0.69–1.30)	1.34 (0.97–1.86)	1.17 (0.85–1.61)
High sensitivity cut-off value^3^	0.135	0.0225	0.0125	0.0225
LR+ (95% CI)	1.12 (0.98–1.28)	1.05 (0.97–1.15)	0.98 (0.88–1.09)	1.0 (0.91–1.09)
LR- (95% CI)	0.49 (0.21–1.14)	0.45 (0.12–1.68)	1.21 (0.46–3.16)	1.05 (0.32–3.49)
High specificity cut-off value^4^	0.685	0.1725	0.57	1.88
LR+ (95% CI)	2.11 (0.96–4.63)	1.05 (0.22–5.06)	0.79 (0.18–3.41)	0.53 (0.10–2.79)
LR- (95% CI)	0.87 (0.75–1.01)	1.0 (0.93–1.07)	1.01 (0.94–1.09)	1.03 (0.96–1.09)

1 From ROC curve

2 From the ROC curve: The level of the index with the highest sensitivity, with at least 50% specificity

3 From the ROC curve: The level of the index with 80% sensitivity or greater

4 From the ROC curve: The level of the index with 80% specificity or greater

LR+ Positive likelihood ratio

LR- Negative likelihood ratio

## Discussion

Our study found a significant association between the Breteau Index and recent transmission of dengue virus at cluster level but not at household level. None of the other indices was associated with dengue infection at household or cluster level. None of the indices was useful as a predictor of dengue infection, although the Breteau Index was marginally better than the other three indices.

There is little consensus in the literature about the association between vector indices and dengue cases or evidence of dengue infection. The 2014 systematic review by Bowman et al found as many studies not reporting association between indices and clinical dengue cases as those finding an association, and the authors noted that the methodology of most studies was weak [20]. Most of the studies analysed routinely collected data about both vector densities and clinical cases of dengue. The review authors suggested it was premature to conclude that surveillance of vector density should be abandoned as a means of predicting dengue outbreaks, but called for more studies to establish the place of such surveillance in local prediction models.

More recently, a study in Indonesia found no significant association between vector indices and dengue transmission (comparing villages with differing numbers of dengue fever cases) [18]. In Peru, Cromwell and colleagues found no significant association between a wide range of vector indices routinely measured cross-sectionally and serological evidence of recent dengue infection among adults, although there were significant associations between longitudinal estimates of vector density and dengue seroconversion. They concluded that routinely collected cross-sectional vector estimates were of limited use for detecting areas at high risk of dengue infection [21].

Two authors have specifically examined the value of dengue vector measurements for predicting the risk of clinical cases of dengue virus infection. In contrast to our findings, they reported that some vector indices could usefully predict areas at high risk of clinical dengue infections. Using data from the 2000 dengue epidemic in Havana, Cuba, Sanchez and colleagues created ROC curves for the Breteau index and found an area under the curve of 71% for predicting neighbourhoods with dengue cases [41]. They subsequently tested the threshold for the Breteau index derived from this study, and found good sensitivity and specificity for predicting neighbourhoods with dengue cases in the 2001 Havana dengue epidemic [42]. The context of these studies is very different from that in Mexico and Nicaragua. Cuba is known to have generally good control of *Aedes aegypti* [43] and the authors point out that their findings relate to areas with low infestation levels. Our mean Breteau index at cluster level was 33, while the mean Breteau index in the Sanchez study, even in case neighbourhoods during the epidemic, was 4.35 [41]. The vector measurements in Cuba came from routine surveillance before, during, and after an outbreak of dengue related to introduction of a new serotype. In Mexico and Nicaragua, where our study took place, dengue is endemic, with all known serotypes in circulation. Authors of a study in a city in Taiwan used routinely collected entomological data and data on meteorological factors over five years to create composite indices which they found were good predictors of dengue cases in parts of the district, with high values of the area under the ROC curve and high sensitivity and specificity [44]. Again, the context of this study is different from our study, in that dengue is non-endemic in Taiwan. The authors recommend development of context-specific predictive models in different countries. The studies in both Cuba and Taiwan considered prediction of clinical cases of dengue [41,42, 44], rather than prediction of dengue infection as in our study.

Our findings of lack of association between vector indices and recent dengue infection and lack of predictive utility of these indices, in both intervention and control clusters, at household and cluster level, are compatible with the impact demonstrated by the Camino Verde trial [22]. The actions to clean up potential mosquito breeding sites taken by people in the trial extended beyond the clusters of about 140 households where we measured the impact on vector indices and on recent infection in children. In most cases the actions covered the whole community and often neighbouring communities as well. The point is that the source of dengue infection is not the individual household or even the cluster, rather it is the whole ecozone. The fact that the *Camino Verde* trial resulted in a reduction in dengue infection, not simply a reduction in vector indices, confirms that the prevention approach–community mobilization based on evidence and autonomous community decisions about actions–is effective. The findings of the present analysis show that control of dengue infection requires working with the whole community, with communities organising themselves to clean up not just individual households but also vacant lots, parks, schools, bus stations, public spaces and streets. Community commitment is needed to undertake prevention activities with local resources [45,46,47].

Our results suggest that the health service prevention strategy of peridomestic fumigation and application of temephos to households around the households of confirmed dengue cases [48], is unlikely to be effective in preventing dengue transmission. Dengue infection can occur in people living in households or neighbourhoods with low or zero vector indices. A previous study found that the individual risk of dengue infection was related to recently visited localities [49].

In our study the entomological indices were high at both time points. They were significantly lower in the intervention clusters, but still high even in these clusters. The entomological data were stable between measurement points with respect to season and type of hatcheries. In our clusters, 99% and 98% of pupal productivity came from conventionally used water storage containers [50]. Barrera reported similar results in Puerto Rico[51]. LaCon et al found different results in Peru [52].

### Strengths and weaknesses

The great majority of reported studies on the association between vector indices and dengue transmission have used clinical cases of dengue fever as the indicator of transmission; Honorio et al in Brazil [53] and Cromwell et al in Peru [21] used dengue serology. Our study used dengue seroconversion of children as the indicator of dengue transmission. Our design allowed us to examine associations between vector indices and dengue infection at both individual household level and cluster level.

We excluded some households from our analysis, in both intervention and control clusters, either because they did not have both entomological reviews or because the children did not provide both saliva samples. If households with higher vector indices were less likely to have both entomological reviews, this could result in under-estimation of vector levels, but we have no reason to think this was the case.

Using saliva testing to detect recent dengue infection may have missed some cases because of its relatively low sensitivity of between 90 and 93 percent. However, saliva testing has the advantage of being non-invasive, increasing the likelihood of obtaining samples [54,55]. We did not measure adult mosquito indices, and it is possible that these indices might be predictive of serological dengue infection. However, there is debate about the association of adult mosquito indices and dengue virus transmission risk [53,56].

## Conclusion

In Mexico and Nicaragua, conventional vector indices were not useful predictors of dengue infection, in either intervention or control clusters of the *Camino Verde* trial of community mobilization for dengue prevention. The findings are compatible with the idea of sources of infection outside the household which were tackled by community action in the *Camino Verde* trial.

## Supporting information

S1 Checklist STROBE Checklist. (DOC)

S1 Data Dataset from Mexico and Nicaragua. (CSV)

S2 Data Coding sheet. (XLS)

S1 Table Correlation of first and second measurements of entomological indices, in 150 clusters in Mexico and Nicaragua. (DOCX)

S2 Table Correlation between entomological indices and serological infection rate at first measurement, in 150 clusters in Mexico and Nicaragua. (DOCX)

S3 Table Associations between vector indices and serological evidence of recent dengue infection in children at the household level, excluding those households that had children infected at the first measurement (15 Units of IgG or more). (DOCX)

S4 Table Associations between vector indices and serological evidence of dengue infection in children at household level, in 150 clusters form Mexico and Nicaragua. (DOCX)

S5 Table Levels of entomological indices after exclusion of households with self-reported dengue. (DOCX)

S6 Table Associations between vector indices and serological evidence of dengue infection in children at household level, excluding households with any self-reported case of dengue. (DOCX)

S7 Table Predictive utility of vector indices at household level (cut-off 0 vs >0) in intervention and control clusters. (DOCX)

S8 Table Predictive utility of vector indices at cluster level in intervention and control clusters. (DOCX)

S1 Fig Frequency distribution of the of the measured Breteau Index ranges at the two measurement times at household level. The blue line represents the initial measurement and red line the final measurement. We exclude zero values in order to better visualize the measured ranges. A Frequency distribution of the Breteau index in all 150 clusters. B. Frequency distribution of the Breteau index in the 75 intervention clusters. C. Frequency distribution of the Breteau index in the 75 control clusters.
(TIF)

S2 Fig Frequency distribution of the container index at the two measurement times at household level. The blue line represents the initial measurement and red line the final measurement. We exclude zero values in order to better visualize the measured ranges. A. Frequency distribution of container index in all 150 clusters. B. Frequency distribution of container index in the 75 intervention clusters. C. Frequency distribution of the container index in the 75 control clusters.
(TIF)

S3 Fig Frequency distribution of the pupa per household index at the two measurement times at household level. The blue line represents the initial measurement and red line the final measurement. We exclude zero values in order to better visualize the measured ranges. A. Frequency distribution of the pupa per household index in all 150 clusters. B. Frequency distribution of the pupa per household index in the 75 intervention clusters. C. Frequency distribution of the pupa per household index in the 75 control clusters.
(TIF)

S4 Fig Frequency distribution of the pupa per container index at the two measurement times at household level. The blue line represents the initial measurement and red line the final measurement. We exclude zero values in order to better visualize the measured ranges. A. Frequency distribution of the pupa per container index in all 150 clusters. B. Frequency distribution of the pupa per container index in the 75 intervention clusters. C. Frequency distribution of the pupa per container index in the 75 control clusters.
(TIF)

S5 Fig Container index and proportion of serology positive households in clusters. A. Scatter plot of container index and proportion of dengue serology positive households in all 150 clusters. B. Scatter plot of container index and proportion of serology positive households in 75 trial intervention clusters. C. Scatter plot of container index and proportion of serology positive households in 75 trial control clusters.
(TIF)

S6 Fig Pupae per household index and proportion of serology positive households in clusters. A. Scatter plot of pupae per household index and proportion of dengue serology positive households in all 150 clusters. B. Scatter plot of pupae per household index and proportion of serology positive households in 75 trial intervention clusters. C. Scatter plot of pupae per household index and proportion of serology positive households in 75 trial control clusters.
(TIF)

S7 Fig Pupae per container index and proportion of serology positive households in clusters. A. Scatter plot of pupae per container index and proportion of dengue serology positive households in all 150 clusters. B. Scatter plot of pupae per container index and proportion of serology positive households in 75 trial intervention clusters. C. Scatter plot of pupae per container index and proportion of serology positive households in 75 trial control clusters.
(TIF)

S8 Fig ROC curve for the Container Index to predict dengue infection at household level. A. ROC curve for Container Index as a predictive test for dengue infection in households in all 150 clusters. B. ROC curve for Container Index as a predictive test for dengue infection in households in 75 trial intervention clusters. C. ROC curve for Container Index as a predictive test for dengue infection in households in 75 trial control clusters.
(TIF)

S9 Fig ROC curve for Pupa per Household Index to predict dengue infection at household level. A. ROC curve for Pupa per Household Index as a predictive test for dengue infection in households in all 150 clusters. B. ROC curve for Pupa per Household Index as a predictive test for dengue infection in households in 75 trial intervention clusters. C. ROC curve for Pupa per Household Index as a predictive test for dengue infection in households in 75 trial control clusters.
(TIF)

S10 Fig ROC curve for Pupa per Container Index to predict dengue infection at household level. A. ROC curve for Pupa per Container Index as a predictive test for dengue infection in households in all 150 clusters. B. ROC curve for Pupa per Container Index as a predictive test for dengue infection in households in 75 trial intervention clusters. C. ROC curve for Pupa per Container Index as a predictive test for dengue infection in households in 75 trial control clusters.
(TIF)

S11 Fig ROC curve for the Container Index to predict dengue infection at cluster level. A. ROC curve for Container Index as a predictive test for dengue infection in all 150 clusters. B. ROC curve for Container Index as a predictive test for dengue infection in 75 trial intervention clusters. C. ROC curve for Container Index as a predictive test for dengue infection in 75 trial control clusters.
(TIF)

S12 Fig ROC curve for Pupa per Household Index to predict dengue infection at household level. A. ROC curve for Pupa per Household Index as a predictive test for dengue infection in all 150 clusters. B. ROC curve for Pupa per Household Index as a predictive test for dengue infection in 75 trial intervention clusters. C. ROC curve for Pupa per Household Index as a predictive test for dengue infection in 75 trial control clusters.
(TIF)

S13 Fig ROC curve for Pupa per Container Index to predict dengue infection at household level. A. ROC curve for Pupa per Container Index as a predictive test for dengue infection in all 150 clusters. B. ROC curve for Pupa per Container Index as a predictive test for dengue infection in 75 trial intervention clusters. C. ROC curve for Pupa per Container Index as a predictive test for dengue infection in 75 trial control clusters.
(TIF)
