# Supplementary material for: Utility of entomological indices for predicting transmission of dengue virus: secondary analysis of data from the Camino Verde trial in Mexico and Nicaragua
Source: PLoS Negl Trop Dis. 2020 Oct 26;14(10):e0008768. doi: 10.1371/journal.pntd.0008768 (PMC7588090; doi:10.1371/journal.pntd.0008768)
Supplement: S1 Table — (DOCX) [file pntd.0008768.s004.docx]

Table S1. Correlation of first and second measurements of entomological indices, in 150 clusters in Mexico and Nicaragua

| Household level | | | |
| --- | --- | --- | --- |
| Index | R | R^2^ | P |
| Breteau index | 0.73 | 0.54 | <0.001 |
| Container index | 0.43 | 0.18 | <0.001 |
| Pupa per household index | 0.66 | 0.44 | <0.001 |
| Pupa per container index | 0.41 | 0.17 | <0.001 |
| Cluster level | | | |
| Index | R | R^2^ | P |
| Breteau index | 0.576 | 0.332 | <0.001 |
| Container index | 0.376 | 0.142 | <0.001 |
| Pupa per household index | 0.301 | 0.091 | <0.001 |
| Pupa per container index | 0.388 | 0.151 | <0.001 |
