# Supplementary material for: Utility of entomological indices for predicting transmission of dengue virus: secondary analysis of data from the Camino Verde trial in Mexico and Nicaragua
Source: PLoS Negl Trop Dis. 2020 Oct 26;14(10):e0008768. doi: 10.1371/journal.pntd.0008768 (PMC7588090; doi:10.1371/journal.pntd.0008768)
Supplement: S2 Table — (DOCX) [file pntd.0008768.s005.docx]

Table S2. Correlation between entomological indices and serological infection rate at first measurement, in 150 clusters in Mexico and Nicaragua

| Index | R | R^2^ | P |
| --- | --- | --- | --- |
| Breteau index | 0.107 | 0.011 | 0.19 |
| Container index | 0.001 | 0.0 | 0.99 |
| Pupa per household index | 0.1 | 0.01 | 0.22 |
| Pupa per container index | 0.119 | 0.014 | 0.15 |
| Intervention group | | | |
| Breteau index | 0.023 | 0.001 | 0.85 |
| Container index | 0.142 | 0.02 | 0.22 |
| Pupa per household index | 0.297 | 0.088 | 0.01 |
| Pupa per container index | 0.28 | 0.08 | 0.01 |
| Control group | | | |
| Breteau | 0.228 | 0.052 | 0.05 |
| Container index | 0.177 | 0.031 | 0.13 |
| Pupa per household index | 0.035 | 0.001 | 0.76 |
| Pupa per container index | 0.075 | 0.006 | 0.52 |
