# Supplementary material for: Utility of entomological indices for predicting transmission of dengue virus: secondary analysis of data from the Camino Verde trial in Mexico and Nicaragua
Source: PLoS Negl Trop Dis. 2020 Oct 26;14(10):e0008768. doi: 10.1371/journal.pntd.0008768 (PMC7588090; doi:10.1371/journal.pntd.0008768)
Supplement: S3 Table — (DOCX) [file pntd.0008768.s006.docx]

Table S3. Associations between vector indices and serological evidence of recent dengue infection in children at the household level, excluding those households that had children infected at the first measurement (15 Units of IgG or more).

| Index | Value | Fraction (%) households with dengue infection serology | | ORa (95% CIca) |
| --- | --- | --- | --- | --- |
|  |  | Negative | Positive^1^ |  |
| BI | 0 | 4038/4558(88.6) | 520/4558(11.4) | 1.12 (0.92-1.36) |
|  | >0 | 440/467(94.2) | 27/467(5.8) |  |
| CI | 0 | 4038/4558(88.6) | 520/4558(11.4) | 1.12 (0.92-1.36) |
|  | >0 | 440/467(94.2) | 27/467(5.8) |  |
| PCI | 0 | 4742/5364(90.5) | 622/5364(11.6) | 1.09 (0.84-1.43) |
|  | >0 | 495/566(87.5) | 71/566(12.5) |  |
| PHI | 0 | 4742/5364(90.5) | 622/5364(11.6) | 1.09 (0.84-1.43) |
|  | >0 | 495/566(87.5) | 71/566(12.5) |  |

^1^ Positive dengue infection serology means at least one child aged 3-9 years old in the household had a doubling of dengue specific antibodies in paired saliva samples

ORa = odds ratio, adjusted for intervention status of the cluster

95%CIca = 95% confidence interval of OR, adjusted for clustering
