# Supplementary material for: Utility of entomological indices for predicting transmission of dengue virus: secondary analysis of data from the Camino Verde trial in Mexico and Nicaragua
Source: PLoS Negl Trop Dis. 2020 Oct 26;14(10):e0008768. doi: 10.1371/journal.pntd.0008768 (PMC7588090; doi:10.1371/journal.pntd.0008768)
Supplement: S4 Table — (DOCX) [file pntd.0008768.s007.docx]

Table S4. Associations between vector indices and serological evidence of dengue infection in children at household level, in 150 clusters form Mexico and Nicaragua

| Index | 1Kendall’s Tau | p |
| --- | --- | --- |
| Breteau index | -0.02 | 0.06 |
| Mean container index | -0.02 | 0.14 |
| Mean pupae per container index | -0.01 | 0.30 |
| Mean pupae per household index | -0.01 | 0.34 |

1Kendall’s tau coefficient

P= p value
