# Supplementary material for: Utility of entomological indices for predicting transmission of dengue virus: secondary analysis of data from the Camino Verde trial in Mexico and Nicaragua
Source: PLoS Negl Trop Dis. 2020 Oct 26;14(10):e0008768. doi: 10.1371/journal.pntd.0008768 (PMC7588090; doi:10.1371/journal.pntd.0008768)
Supplement: S5 Table — (DOCX) [file pntd.0008768.s008.docx]

Table S5. Levels of entomological indices after exclusion of households with self-reported dengue

| Index | Both groups | | Intervention group | | Control group | |
| --- | --- | --- | --- | --- | --- | --- |
|  | First measurement  N= 5949 | Second measurement  N= 5949 | First measurement  N= 3091 | Second measurement  N=3091 | First measurement  N=2858 | Second measurement  N= 2858 |
| BI | 0.274 | 0.235 | 0.214 | 0.172 | 0.34 | 0.303 |
| CI | 0.075 | 0.06 | 0.054 | 0.043 | 0.097 | 0.079 |
| PPHI | 0.598 | 0.562 | 0.387 | 0.31 | 0.828 | 0.835 |
| PPCI | 0.163 | 0.143 | 0.09 | 0.067 | 0.243 | 0.225 |
