# Supplementary material for: Utility of entomological indices for predicting transmission of dengue virus: secondary analysis of data from the Camino Verde trial in Mexico and Nicaragua
Source: PLoS Negl Trop Dis. 2020 Oct 26;14(10):e0008768. doi: 10.1371/journal.pntd.0008768 (PMC7588090; doi:10.1371/journal.pntd.0008768)
Supplement: S6 Table — (DOCX) [file pntd.0008768.s009.docx]

Table S6. Associations between vector indices and serological evidence of dengue infection in children at household level, excluding households with any self-reported case of dengue

| Index | Value | Fraction (%) households with serological infection: | | ORa (95% CIca) |
| --- | --- | --- | --- | --- |
|  |  | Negative | Positive1 |  |
| BI | 0 | 4009/4565(87.8) | 556/4009(12.2) | 1.14 (0.89-1.43) |
|  | >0 | 1195/1384(86.3) | 189/1384(13.7) |  |
| CI | 0 | 4009/4565(87.8) | 556/4009(12.2) | 1.14 (0.89-1.43) |
|  | >0 | 1195/1384(86.3) | 189/1384(13.7) |  |
| PCI | 0 | 4704/5369(87.6) | 665/5369(12.4) | 1.13 (0.79-1.62) |
|  | >0 | 665/5369(86.2) | 80/580(13.8) |  |
| PHI | 0 | 4704/5369(87.6) | 665/5369(12.4) | 1.13 (0.79-1.62) |
|  | >0 | 665/5369(86.2) | 80/580(13.8) |  |

1 Positive serology means at least one child aged 3-9 years old in the household had a doubling of dengue specific antibodies in paired saliva samples

ORa = odds ratio, adjusted for intervention status of the cluster

95%CIca = 95% confidence interval of OR, adjusted for clustering
