# Supplementary material for: Utility of entomological indices for predicting transmission of dengue virus: secondary analysis of data from the Camino Verde trial in Mexico and Nicaragua
Source: PLoS Negl Trop Dis. 2020 Oct 26;14(10):e0008768. doi: 10.1371/journal.pntd.0008768 (PMC7588090; doi:10.1371/journal.pntd.0008768)
Supplement: S7 Table — (DOCX) [file pntd.0008768.s010.docx]

Table S7. Predictive utility of vector indices at household level (cut-off 0 vs >0) in intervention and control clusters

| **Households in 75 intervention clusters** | | | | |
| --- | --- | --- | --- | --- |
|  | BI | CI | PPC | PPH |
| Area under curve^1^ | 0.51  (0.48-0.54) | 0.51  (0.48-0.54) | 0.50  (0.47-0.53) | 0.50  (0.47-0.53) |
| LR+ | 1.08  (0.87-1.34) | 1.08  (0.87-1.34) | 1.09  (0.74-1.60) | 1.09  (0.74-1.60) |
| LR- | 0.98  (0.93-1.04) | 0.98  (0.93-1.04) | 0.99  (0.96-1.02) | 0.99  (0.96-1.02) |
| **Households in 75 control clusters** | | | | |
|  | BI | CI | PPC | PPH |
| Area under curve^1^ | 0.51  (0.48-0.54) | 0.51  (0.48-0.54) | 0.50  (0.48-0.53) | 0.50  (0.48-0.53) |
| LR+ | 1.05  (0.90-1.22) | 1.05  (0.90-1.22) | 1.04  (0.81-1.34) | 1.04  (0.81-1.34) |
| LR- | 0.98  (0.92-1.05) | 0.98  (0.93-1.04) | 0.99  (0.96-1.03) | 0.99  (0.96-1.03) |

^1^ From ROC curve

LR+ Positive likelihood ratio

LR- Negative likelihood ratio
