# Supplementary material for: Utility of entomological indices for predicting transmission of dengue virus: secondary analysis of data from the Camino Verde trial in Mexico and Nicaragua
Source: PLoS Negl Trop Dis. 2020 Oct 26;14(10):e0008768. doi: 10.1371/journal.pntd.0008768 (PMC7588090; doi:10.1371/journal.pntd.0008768)
Supplement: S8 Table — (DOCX) [file pntd.0008768.s011.docx]

Table S8. Predictive utility of vector indices at cluster level in intervention and control clusters

| **Intervention clusters** | | | | |
| --- | --- | --- | --- | --- |
|  | Breteau | Container | PPC | PPH |
| Area under curve^1^ | 0.62  (0.5-0.75) | 0.54  (0.41-0.68) | 0.42  (0.29-0.55) | 0.41  (0.28-0.54) |
| Optimal cut-off value^2^ | 0.2125 | 0.068 | 0.2525 | 0.2775 |
| LR+ | 1.2  (0.81-1.77) | 1.08  (0.65-1.79) | 0.80  (0.51-1.25) | 0.80  (0.48-1.33) |
| LR- | 0.78  (0.46-1.34) | 0.94  (0.63-1.41) | 1.28  (0.80-2.05) | 1.20  (0.80-1.81) |
| High sensitivity cut-off value^3^ | 0.0775 | 0.0225 | 0.035 | 0.0175 |
| LR+ | 1.11  (0.93-1.32) | 1.08  (0.93-1.24) | 1.01  (0.86-1.2) | 0.94  (0.76-1.16) |
| LR- | 0.49  (0.14-1.75) | 0.46  (0.09-2.21) | 0.91  (0.27-3.14) | 1.33  (0.49-3.59) |
| High specificity cut-off value^4^ | 0.43 | 0.1175 | 0.91 | 0.50 |
| LR+ | 3.14  (1.1-8.99) | 1.52  (0.37-6.38) | 0.23  (0.03-1.86) | 0  (-) |
| LR- | 0.76  (0.60-0.98) | 0.96  (0.83-1.11) | 1.11  (0.97-1.26) | 1.05  (0.99-1.13) |
| **Control clusters** | | | | |
| Area under curve^1^ | 0.63  (0.51-0.76) | 0.50  (0.37-0.64) | 0.48  (0.35-0.61) | 0.43  (0.30-0.56) |
| Optimal cut-off value^2^ | 0.2725 | 0.0875 | 0.56 | 0.165 |
| LR+ | 1.08  (0.84-1.4) | 1.09  (0.68-1.75) | 1.16  (0.71-1.88) | 0.92  (0.55-1.53) |
| LR- | 0.78  (0.35-1.75) | 0.93  (0.60-1.43) | 0.88  (0.58-1.35) | 1.07  (0.72-1.60) |
| High sensitivity cut-off value^3^ | 0.0775 | 0.03 | 0.0725 | 0.02 |
| LR+ | 0.95  (0.85-1.05) | 0.95  (0.83-1.08) | 0.97  (0.82-1.15) | 0.95  (0.81-1.11) |
| LR- | 2.92  (0.32-26.83) | 1.95  (0.38-10.0) | 1.22  (0.35-4.18) | 1.62  (0.42-6.31) |
| High specificity cut-off value^4^ | 0.57 | 0.175 | 1.5 | 0.7625 |
| LR+ | 3.57  (1.08-11.78) | 0.97  (0.14-6.56) | 0.58  (0.15-2.27) | 0.97  (0.14-6.56) |
| LR- | 0.77  (0.62-0.97) | 1.0  (0.90-1.12) | 1.06  (0.91-1.25) | 1.0  (0.90-1.12) |

^1^ From ROC curve

^2^ From the ROC curve: The level of the index with the highest sensitivity, with at least 50% specificity

^3^ From the ROC curve: The level of the index with 80% sensitivity or greater

^4^ From the ROC curve: The level of the index with 80% specificity or greater

LR+ Positive likelihood ratio

LR- Negative likelihood ratio
