# Supplementary figures and images for: Utility of entomological indices for predicting transmission of dengue virus: secondary analysis of data from the Camino Verde trial in Mexico and Nicaragua
Source: PLoS Negl Trop Dis. 2020 Oct 26;14(10):e0008768. doi: 10.1371/journal.pntd.0008768 (PMC7588090; doi:10.1371/journal.pntd.0008768)

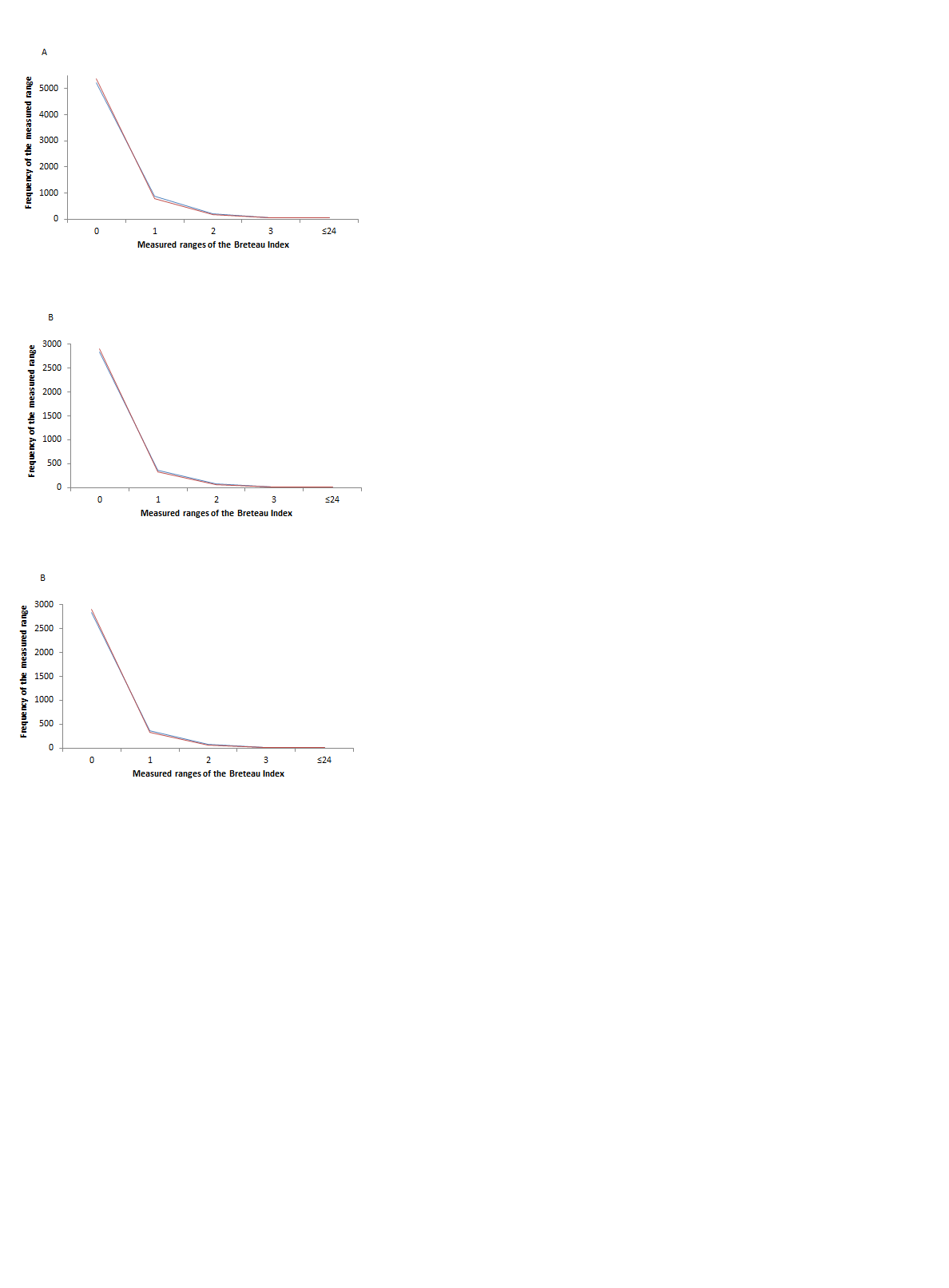

Supplement: S1 Fig — The blue line represents the initial measurement and red line the final measurement. We exclude zero values in order to better visualize the measured ranges. A Frequency distribution of the Breteau index in all 150 clusters. B. Frequency distribution of the Breteau index in the 75 intervention clusters. C. Frequency distribution of the Breteau index in the 75 control clusters. (TIF) [file pntd.0008768.s012.tif]

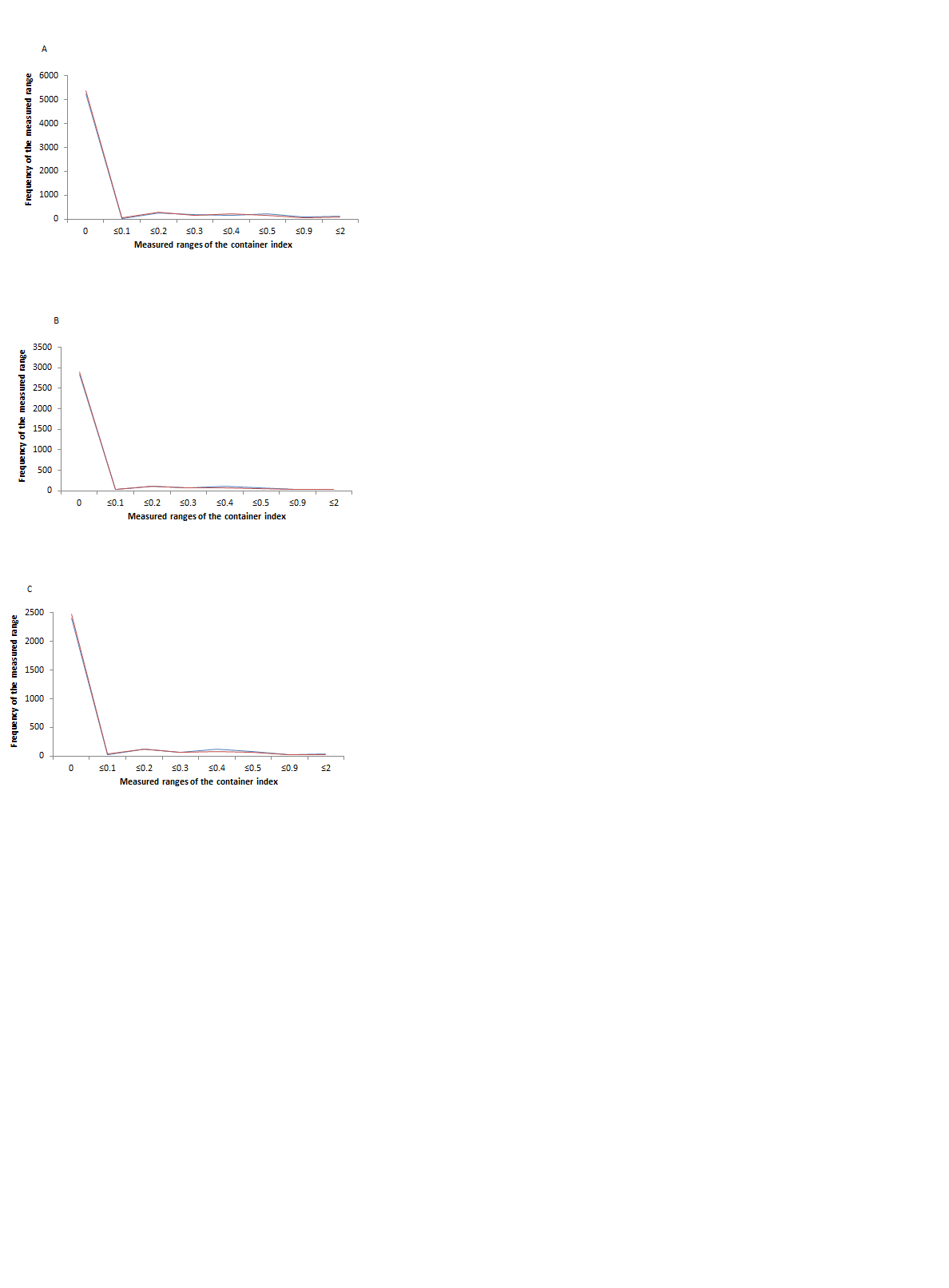

Supplement: S2 Fig — The blue line represents the initial measurement and red line the final measurement. We exclude zero values in order to better visualize the measured ranges. A. Frequency distribution of container index in all 150 clusters. B. Frequency distribution of container index in the 75 intervention clusters. C. Frequency distribution of the container index in the 75 control clusters. (TIF) [file pntd.0008768.s013.tif]

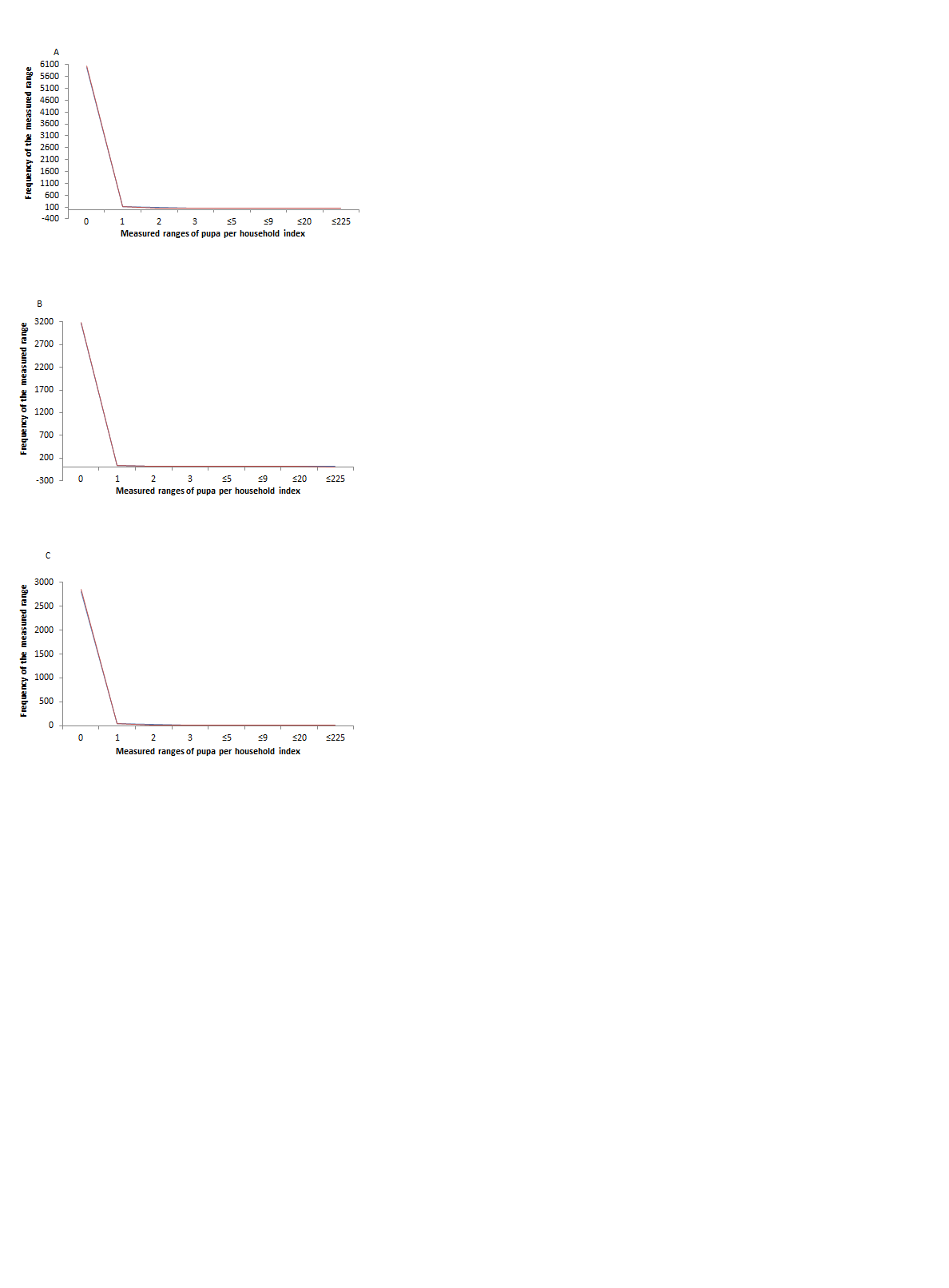

Supplement: S3 Fig — The blue line represents the initial measurement and red line the final measurement. We exclude zero values in order to better visualize the measured ranges. A. Frequency distribution of the pupa per household index in all 150 clusters. B. Frequency distribution of the pupa per household index in the 75 intervention clusters. C. Frequency distribution of the pupa per household index in the 75 control clusters. (TIF) [file pntd.0008768.s014.tif]

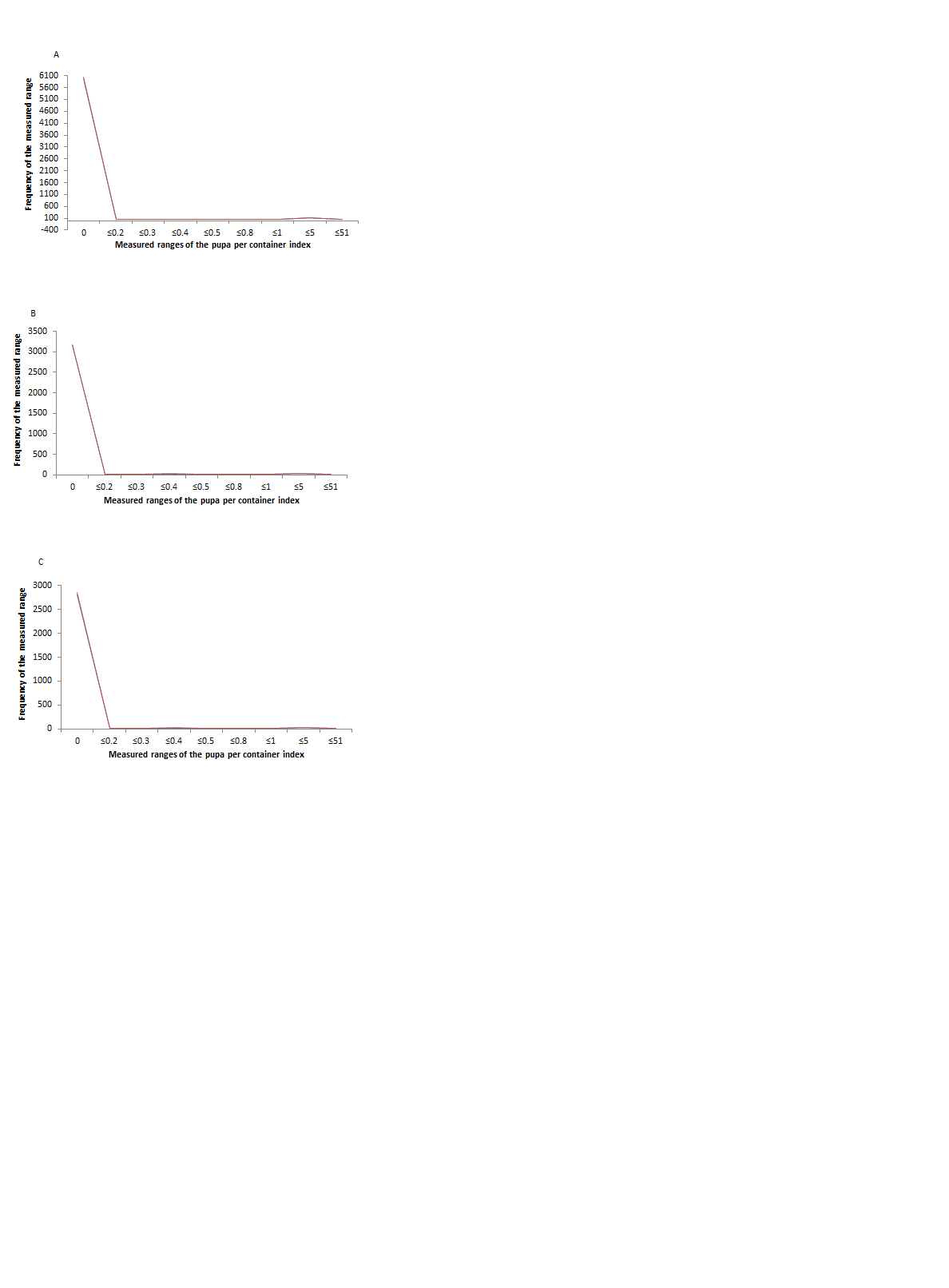

Supplement: S4 Fig — The blue line represents the initial measurement and red line the final measurement. We exclude zero values in order to better visualize the measured ranges. A. Frequency distribution of the pupa per container index in all 150 clusters. B. Frequency distribution of the pupa per container index in the 75 intervention clusters. C. Frequency distribution of the pupa per container index in the 75 control clusters. (TIF) [file pntd.0008768.s015.tif]

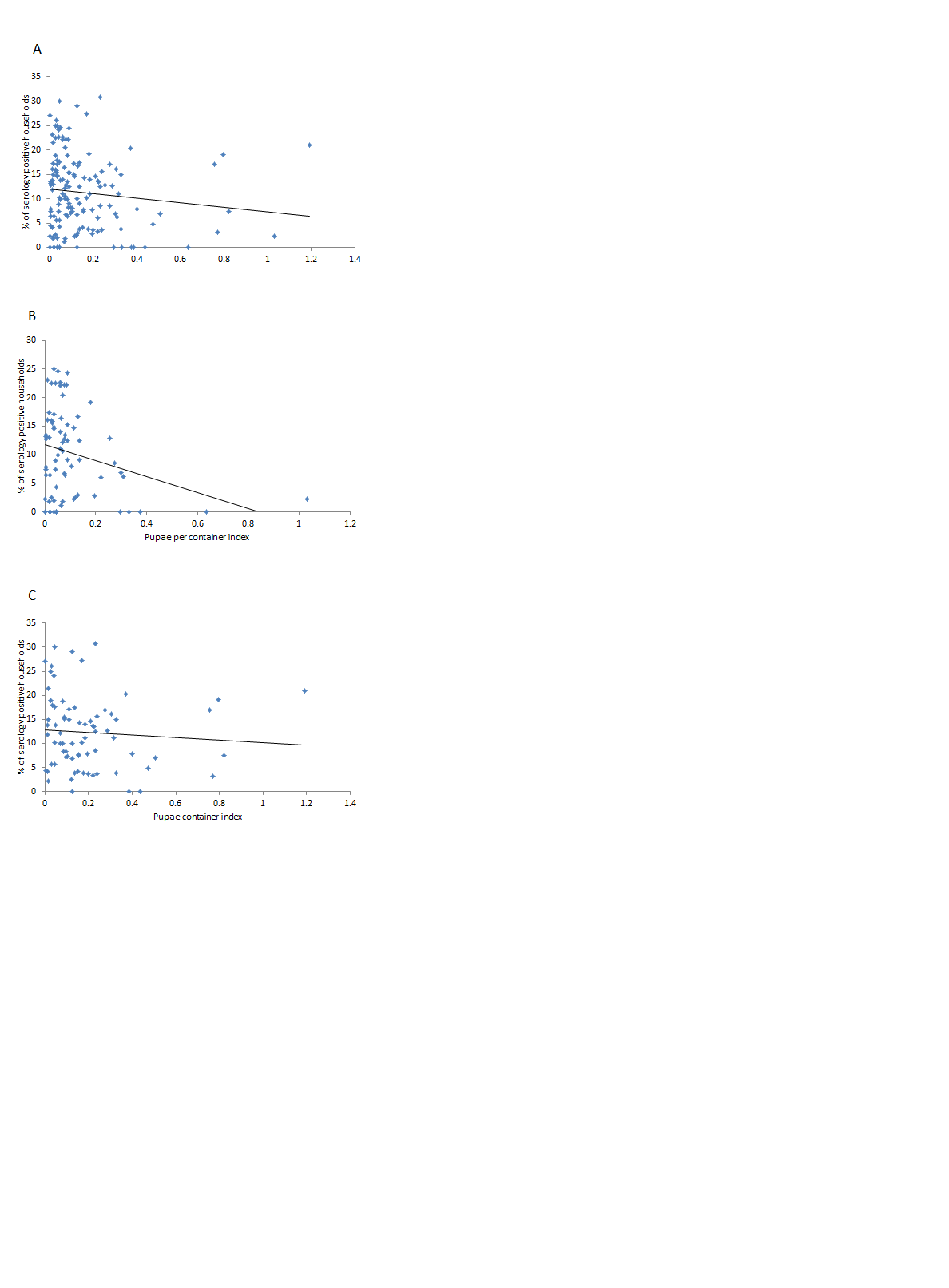

Supplement: S5 Fig — A. Scatter plot of container index and proportion of dengue serology positive households in all 150 clusters. B. Scatter plot of container index and proportion of serology positive households in 75 trial intervention clusters. C. Scatter plot of container index and proportion of serology positive households in 75 trial control clusters. (TIF) [file pntd.0008768.s016.tif]

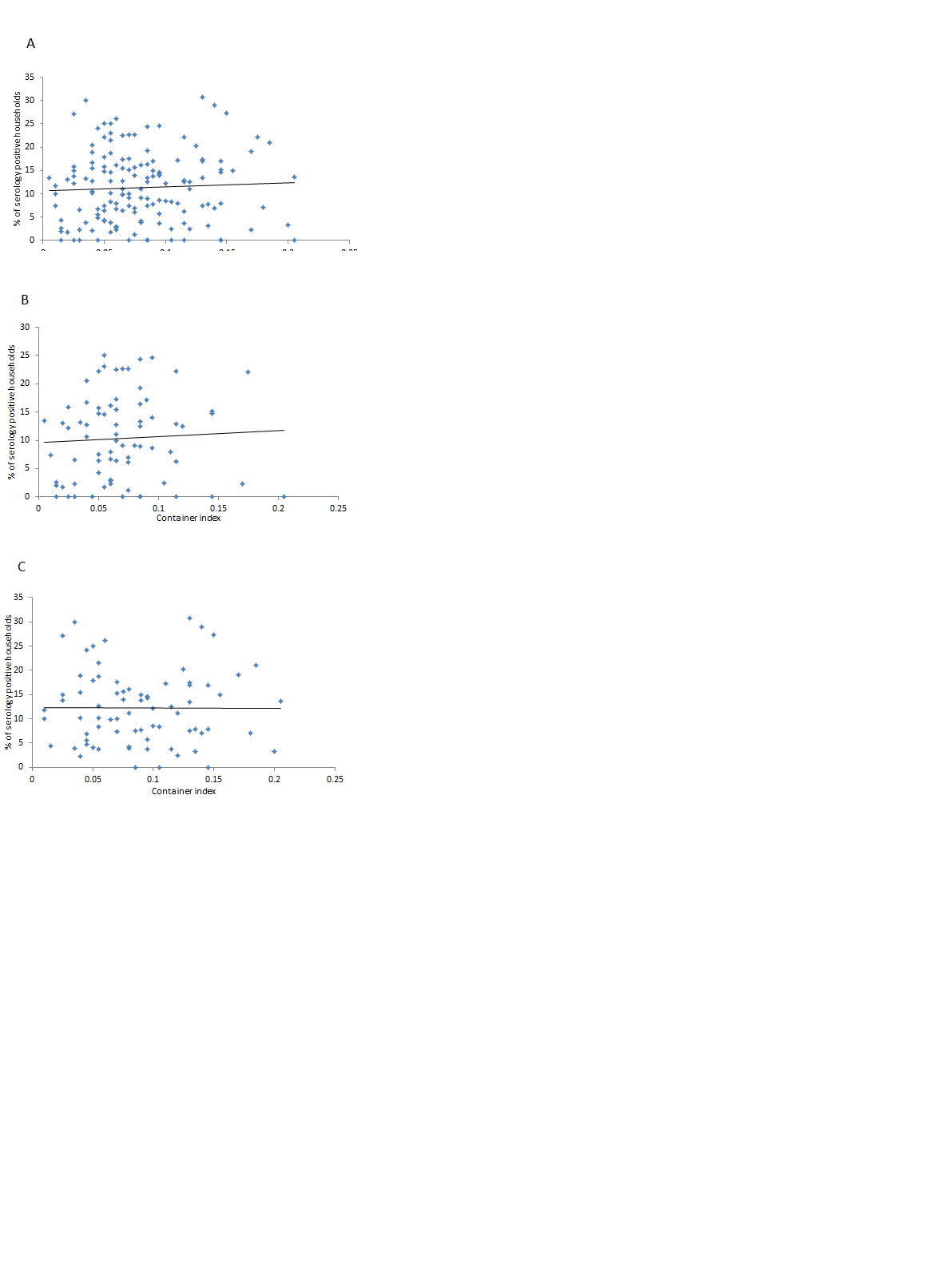

Supplement: S6 Fig — A. Scatter plot of pupae per household index and proportion of dengue serology positive households in all 150 clusters. B. Scatter plot of pupae per household index and proportion of serology positive households in 75 trial intervention clusters. C. Scatter plot of pupae per household index and proportion of serology positive households in 75 trial control clusters. (TIF) [file pntd.0008768.s017.tif]

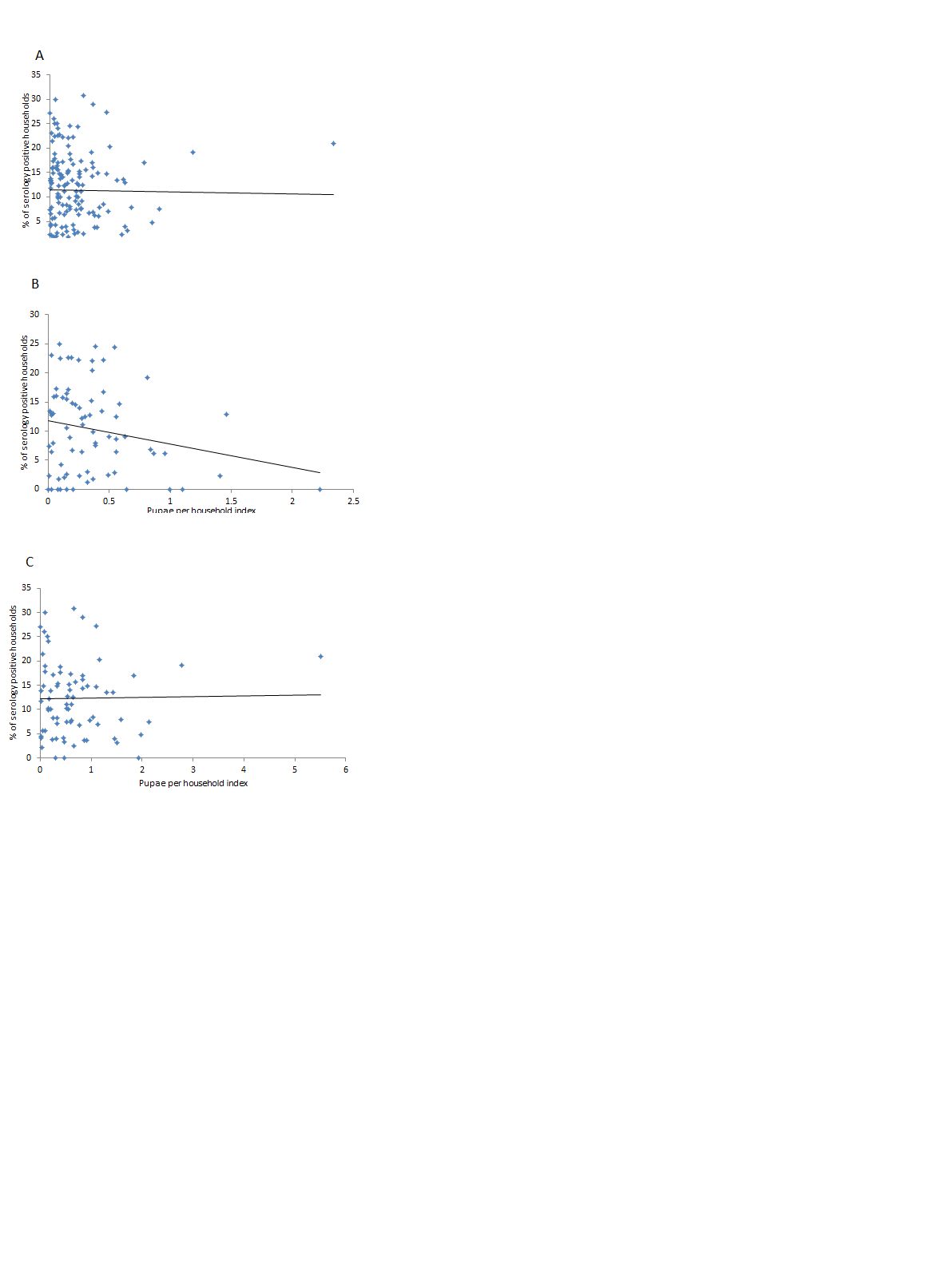

Supplement: S7 Fig — A. Scatter plot of pupae per container index and proportion of dengue serology positive households in all 150 clusters. B. Scatter plot of pupae per container index and proportion of serology positive households in 75 trial intervention clusters. C. Scatter plot of pupae per container index and proportion of serology positive households in 75 trial control clusters. (TIF) [file pntd.0008768.s018.tif]

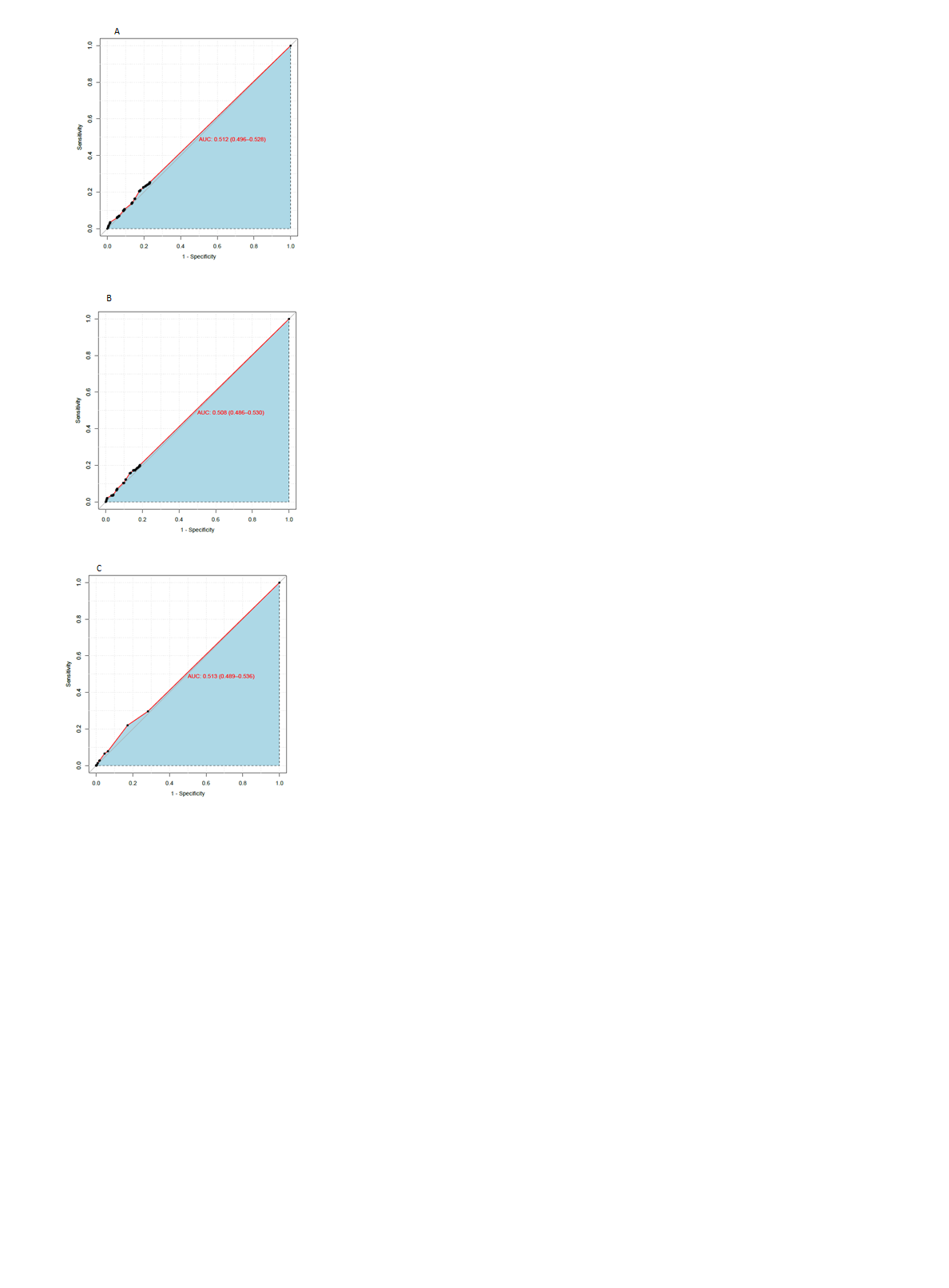

Supplement: S8 Fig — A. ROC curve for Container Index as a predictive test for dengue infection in households in all 150 clusters. B. ROC curve for Container Index as a predictive test for dengue infection in households in 75 trial intervention clusters. C. ROC curve for Container Index as a predictive test for dengue infection in households in 75 trial control clusters. (TIF) [file pntd.0008768.s019.tif]

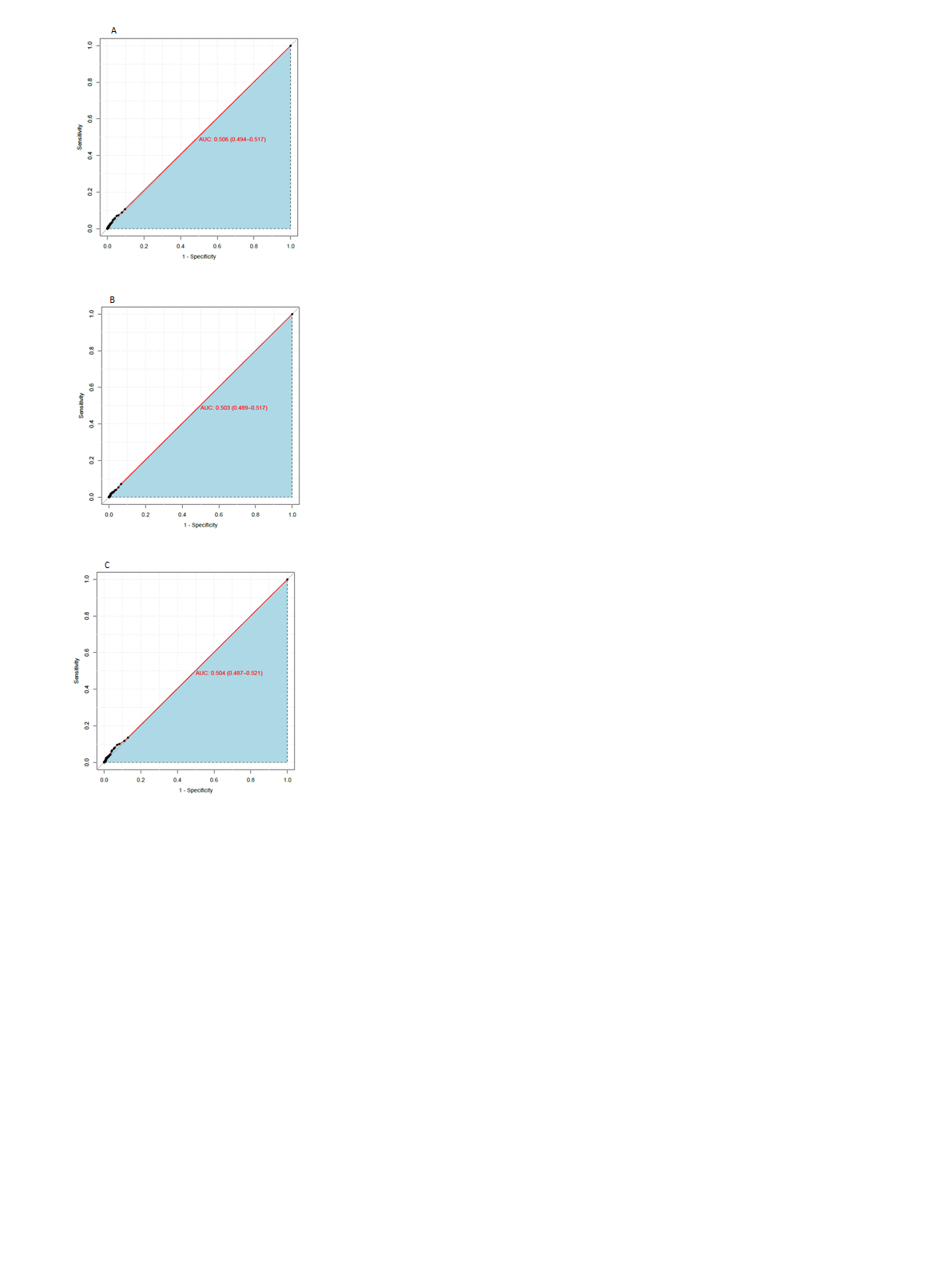

Supplement: S9 Fig — A. ROC curve for Pupa per Household Index as a predictive test for dengue infection in households in all 150 clusters. B. ROC curve for Pupa per Household Index as a predictive test for dengue infection in households in 75 trial intervention clusters. C. ROC curve for Pupa per Household Index as a predictive test for dengue infection in households in 75 trial control clusters. (TIF) [file pntd.0008768.s020.tif]

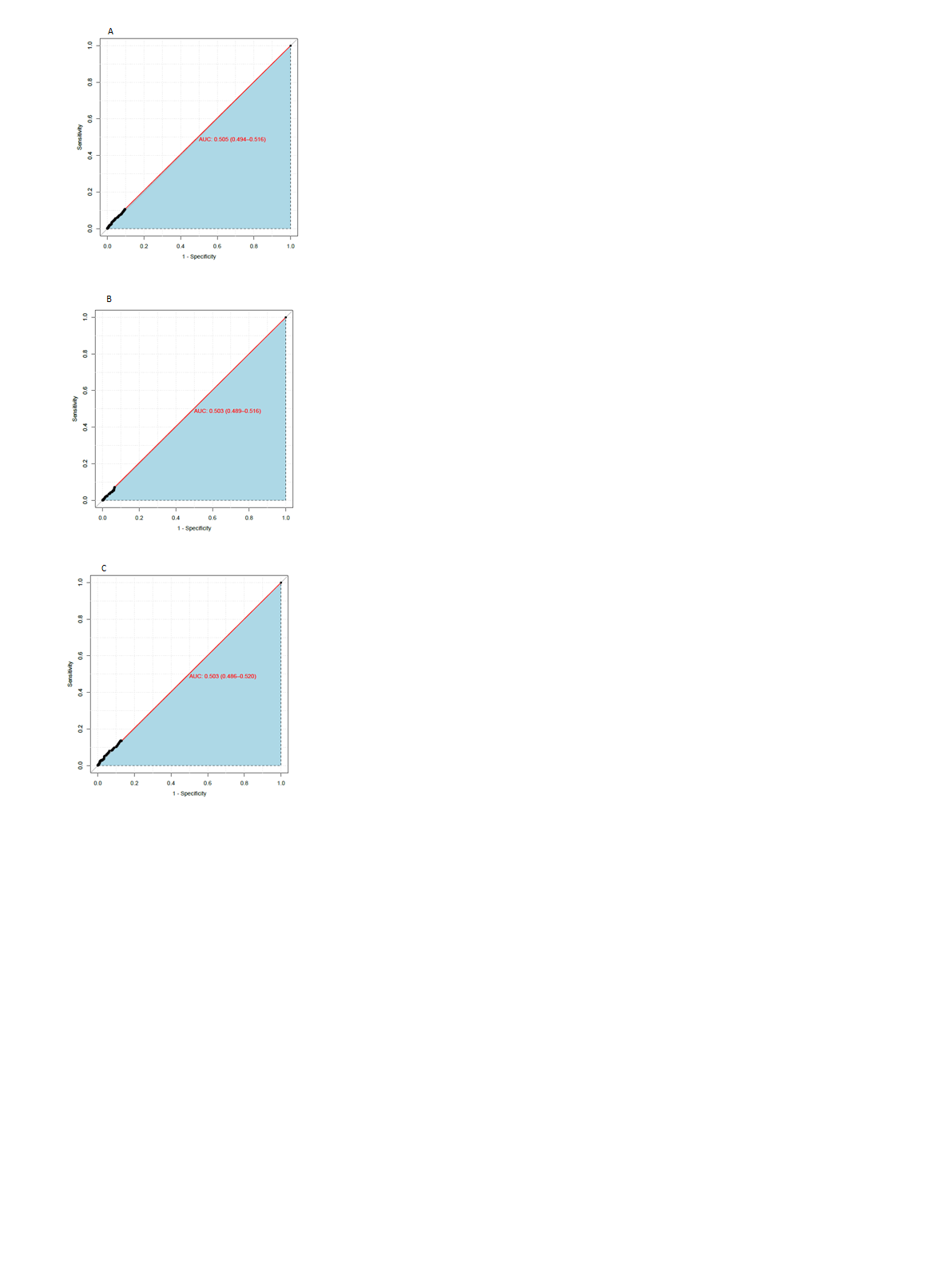

Supplement: S10 Fig — A. ROC curve for Pupa per Container Index as a predictive test for dengue infection in households in all 150 clusters. B. ROC curve for Pupa per Container Index as a predictive test for dengue infection in households in 75 trial intervention clusters. C. ROC curve for Pupa per Container Index as a predictive test for dengue infection in households in 75 trial control clusters. (TIF) [file pntd.0008768.s021.tif]

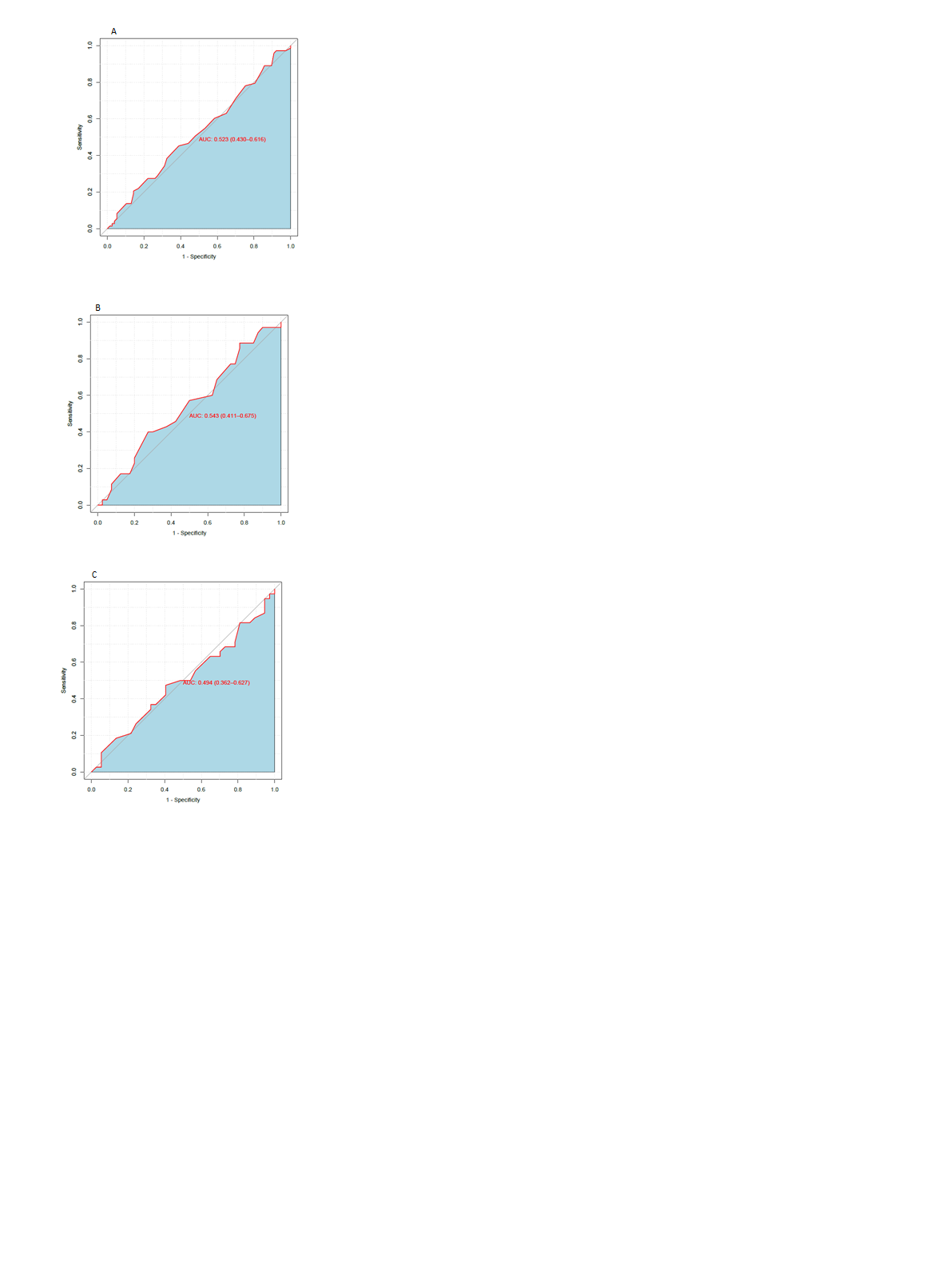

Supplement: S11 Fig — A. ROC curve for Container Index as a predictive test for dengue infection in all 150 clusters. B. ROC curve for Container Index as a predictive test for dengue infection in 75 trial intervention clusters. C. ROC curve for Container Index as a predictive test for dengue infection in 75 trial control clusters. (TIF) [file pntd.0008768.s022.tif]

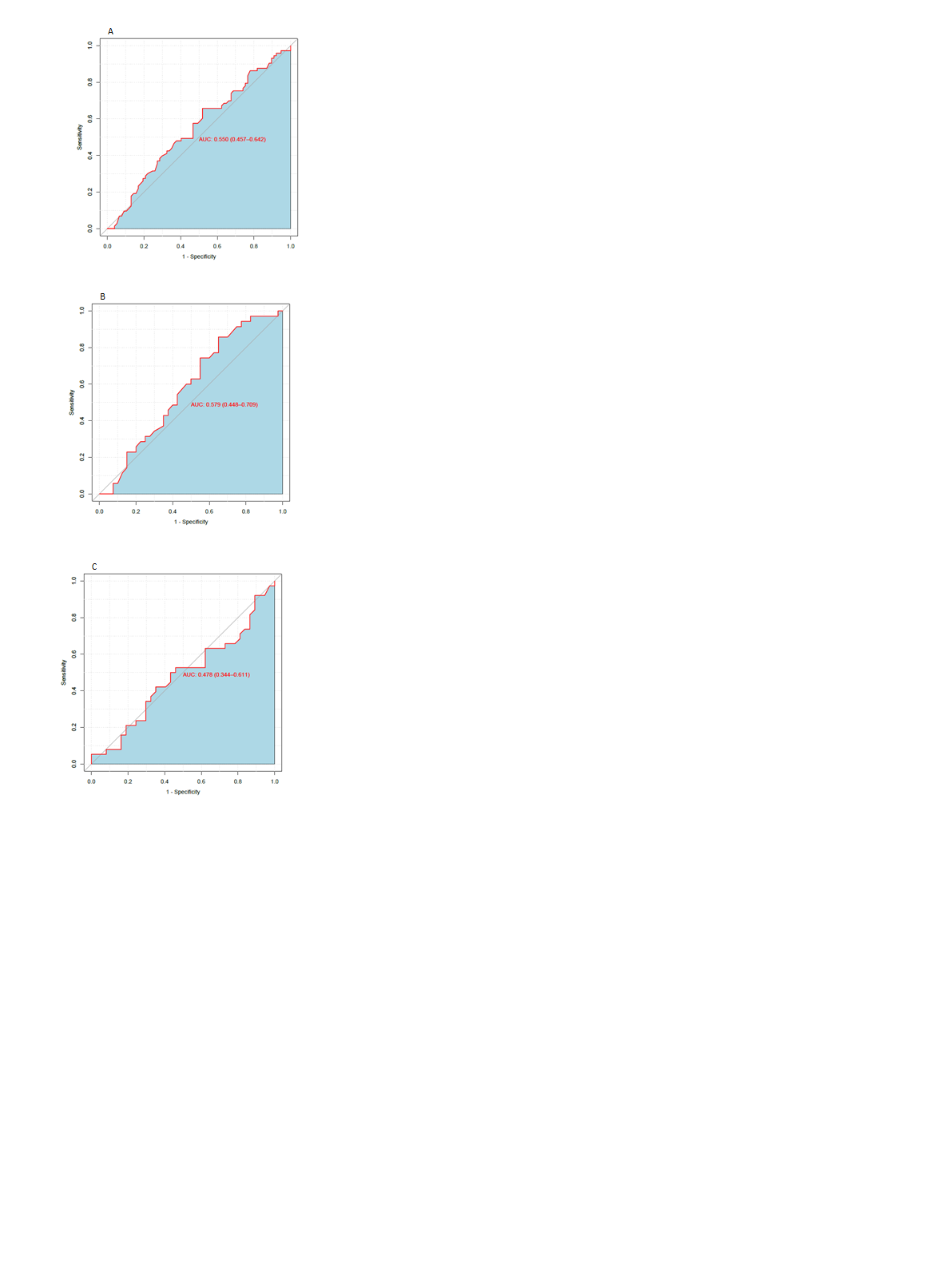

Supplement: S12 Fig — A. ROC curve for Pupa per Household Index as a predictive test for dengue infection in all 150 clusters. B. ROC curve for Pupa per Household Index as a predictive test for dengue infection in 75 trial intervention clusters. C. ROC curve for Pupa per Household Index as a predictive test for dengue infection in 75 trial control clusters. (TIF) [file pntd.0008768.s023.tif]

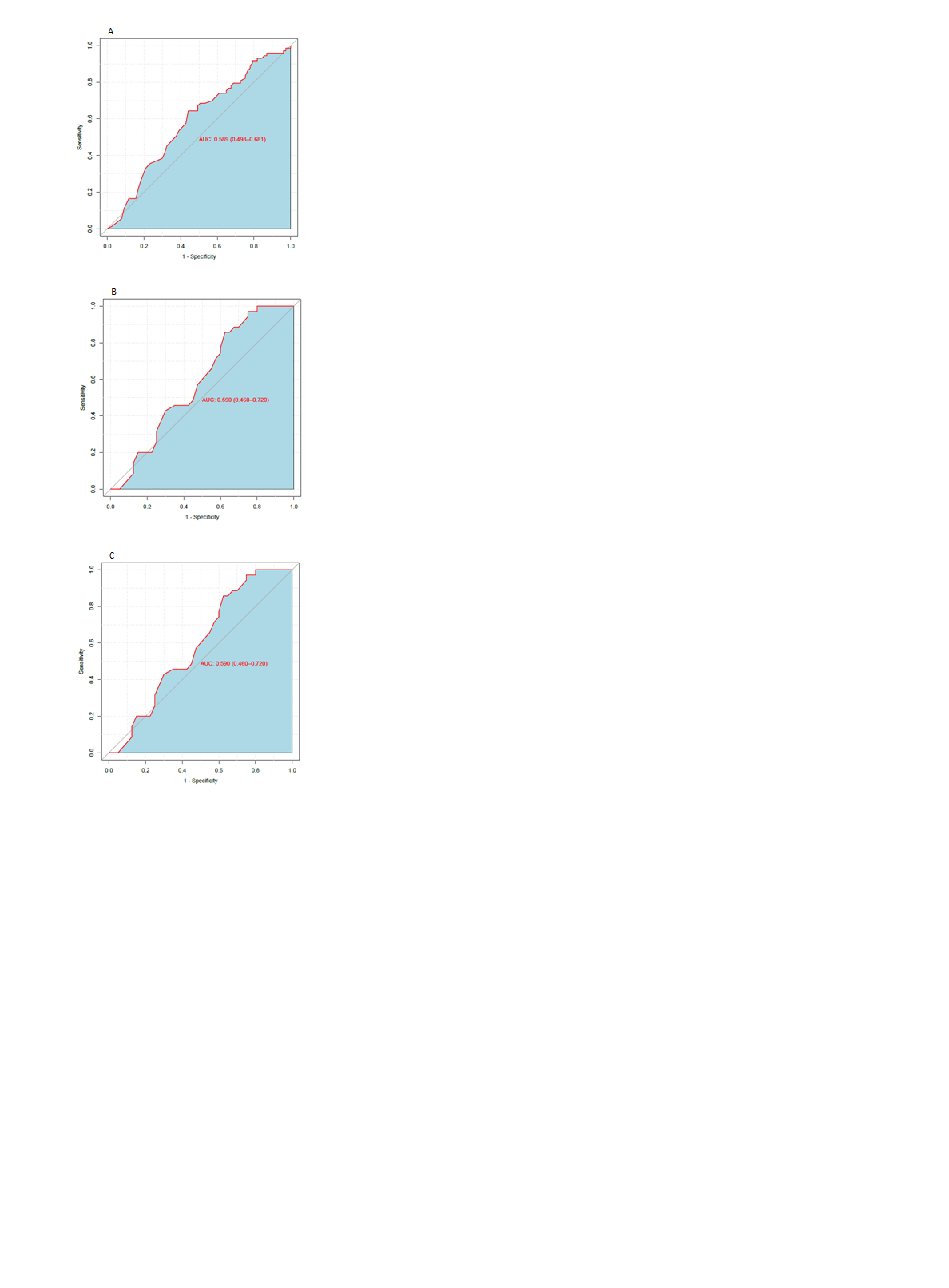

Supplement: S13 Fig — A. ROC curve for Pupa per Container Index as a predictive test for dengue infection in all 150 clusters. B. ROC curve for Pupa per Container Index as a predictive test for dengue infection in 75 trial intervention clusters. C. ROC curve for Pupa per Container Index as a predictive test for dengue infection in 75 trial control clusters. (TIF) [file pntd.0008768.s024.tif]
